# Supplementary material for: An open source tool to infer epidemiological and immunological dynamics from serological data: serosolver
Source: PLoS Comput Biol. 2020 May 4;16(5):e1007840. doi: 10.1371/journal.pcbi.1007840 (PMC7241836; doi:10.1371/journal.pcbi.1007840)
Supplement: S1 Text — (PDF) [file pcbi.1007840.s001.pdf]

An open source tool to infer epidemiological and immunological dynamics from serological data: serosolver

Supporting Text 1: general statistical framework and derivation of the infection history priors

Contents

1 Motivation **2**

2 Derivation of the full model **2**

2.1 Observations of latent antibody levels 2
2.2 Generation of latent antibody levels from infections 2
2.3 Infection history model 3
2.4 Full model 3

3 Intuitive prior **4**

4 Priors in *serosolver* **5**

4.1 Prior 1, hyper-prior on per-time infection probability 5
4.1.1 Prior on number of lifetime infections 6
4.2 Prior 2, beta prior on the probability of infection in each time period j 6
4.2.1 Prior on number of lifetime infections 9
4.3 Prior 3, beta-binomial prior on the total number of lifetime infections 9
4.3.1 Prior on attack rates 10
4.4 Prior 4, beta prior on the probability of any infection event 11

5 Choice of prior **11**

6 Appendix **18**

6.1 Proposal algorithm under prior version 1, hyper-prior on probability of infection 18
6.2 Proposal algorithm under prior version 2, beta prior on attack rates 19
6.3 Proposal algorithm under prior version 3, beta prior on per-individual infection probability 20
6.4 Proposal algorithm under prior version 4, beta prior on overall probability of infection 21

1 Motivation

Here, we provide further details on the assumptions and priors required to perform Bayesian inference of infection histories with a model considering past infection outcomes of a population as a matrix of latent features. This also serves as a reference guide to understand the impact of the different priors on inferred infection histories and attack rates in the accompanying *serosolver* package. Although the framework is motivated by and developed for influenza, the antibody kinetics and infection history models are conceptually separate. Deriving the full model is therefore first framed as a general statistical challenge: what is a good model to represent multiple hidden infection states? As in the main text, vectors are represented in bold, capital letters represent random variables and lower case letters represent values of random variables.

2 Derivation of the full model

Infection events are not observed directly. Rather, exposure to antigens lead to the production of antibodies that undergo longitudinal and cross-reactive kinetics, which can be observed by taking serum samples. Note that we use the term 'infection' to mean any exposure that elicits an antibody boost, though model extensions could distinguish between different types of exposure (e.g. vaccination). The system we wish to describe is therefore split into three conceptual levels: (i) the set of serological data (antibody titres) that we observe; (ii) the true underlying (latent) antibody levels that gave rise to these observations; and (iii) the underlying set of infections or exposures that gave rise to these true antibody levels. In this section, we build the full *serosolver* model through these three levels.

2.1 Observations of latent antibody levels

Measurements of true underlying antibody levels are subject to noise in the observation process. This may arise from observation error, assay preparation error, sample collection variability etc. This observation process may be generically defined as:

$$\boldsymbol{Y_{i,t}} \sim e(\boldsymbol{X_{i,t}}, \boldsymbol{\Theta}) \tag{1}$$

where $e(\boldsymbol{X_{i,t}}, \boldsymbol{\Theta})$ represents the stochastic process of generating observations from the latent antibody levels at time t, $\boldsymbol{X_{i,t}}$; $\boldsymbol{Y_{i,t}}$ represents the set of observed antibody titres; and $\boldsymbol{\Theta}$ represents the vector of all model parameters e.g. variance for a Gaussian observation model. The likelihood of observing $\boldsymbol{Y_{i,t}}$ given $\boldsymbol{X_{i,t}}$ and $\boldsymbol{\Theta}$ is defined as $P(\boldsymbol{Y_{i,t}}|\boldsymbol{X_{i,t}}, \boldsymbol{\Theta})$.

2.2 Generation of latent antibody levels from infections

An individual's latent antibody levels, $\boldsymbol{X_{i,t}}$, at time t are generated as a function of all infections prior to or during time t ($j \leq t$) and the antibody kinetics parameters, $\boldsymbol{\Theta}$. For example, each infection may lead to a boost in antibody titres that accumulates with each successive infection.

These unobserved infection events are modelled as latent binary states, $Z_{i,j}$. Each latent infection state is the outcome of a single Bernoulli trial, where $z_{i,j} = 1$ indicates that individual i was infected with the strain circulating during discrete time period j, and $z_{i,j} = 0$ indicates that they were not. We assume that there is only one strain that circulates during each discrete time period, and j therefore refers to both the time period j and the strain that circulated during that time. Strains may be antigenically identical in each discrete time period or exhibit antigenic variation.

The antibody kinetics model describing the generation of latent antibody levels given the vector of unobserved infection states is given as:

$$\boldsymbol{X_{i,t}} = g(Z_{i,1}, Z_{i,2}, \ldots, Z_{i,j\leq t}, \boldsymbol{\Theta}) \tag{2}$$

where g may be any arbitrary model function and $Z_{i,j\leq t}$ denotes the last infection that could have occurred at or before discrete time period t. In the main text model, the model g captures deterministic antibody boosting, waning, and cross-reactivity. In this example, $\boldsymbol{X_{i,t}} = [X_{i,1,t}, X_{i,2,t}, \ldots, X_{i,j,t}]$, represents a vector of latent titres against each strain that circulated during each possible exposure time j. $\boldsymbol{Z_i} = [Z_{i,1}, Z_{i,2}, \ldots, Z_{i,j\leq t}]$ to represent the vector of infection states that could have been realised at or before the serum sample taken at time t.

The likelihood of generating latent antibody levels, $\boldsymbol{X_{i,t}}$, given the vector of latent infection states, $\boldsymbol{Z_i}$ is defined as $P(\boldsymbol{X_{i,j}}|\boldsymbol{Z_i}, \boldsymbol{\Theta})$. Note that when g is deterministic, $P(\boldsymbol{X_{i,j}}|\boldsymbol{Z_i}, \boldsymbol{\Theta}) = 1$ for all values of $\boldsymbol{Z_i}$ and $\boldsymbol{\Theta}$.

2.3 Infection history model

If we consider a system with n individuals who may be infected once in each of m distinct time periods, then there are nm possible infection events. We are interested in jointly inferring the posterior distributions outcomes of each of these nm infection events and a set of antibody kinetics parameters using serological data. In terms of inference, only $\boldsymbol{Y}$ is observed, so we must infer (or augment) the values of $\boldsymbol{Z}$ as latent features. We must therefore define a model for the generation of $\boldsymbol{Z}$ defined broadly as $P(\boldsymbol{Z})$ here. Throughout this supplement, we discuss how the model for $P(\boldsymbol{Z})$ may be chosen to capture different assumptions about the epidemiological process that generates infections.

2.4 Full model

Through combining the three levels, the full inference problem can be framed as estimating the following joint posterior distribution:

$$P(\boldsymbol{Z}, \boldsymbol{X}, \boldsymbol{\theta}|\boldsymbol{Y}) = \prod_{i=1}^{n} \Big(\prod_{t\in t_i} \underbrace{P(\boldsymbol{Y_{i,t}}|\boldsymbol{X_{i,t}}, \boldsymbol{\Theta})}_{\text{Observation model}} \Big) \prod_{j=j_{min}}^{j_{max}} \underbrace{P(\boldsymbol{X_{i,j}}|\boldsymbol{Z_i}, \boldsymbol{\Theta})}_{\text{Antibody kinetics model}} \underbrace{P(Z_{i,j})}_{\text{Infection history model}} P(\boldsymbol{\Theta}) \tag{3}$$

where t_i represents the set of serum sampling times for individual i; j_{min} to j_{max} represents the range of times over which individuals may be infected; and $\boldsymbol{\Theta}$ is the vector of antibody kinetics parameters describing the link between $\boldsymbol{Z}$ and $\boldsymbol{Y}$. $P(\boldsymbol{\Theta})$ can be represented by standard prior distributions.

$P(\boldsymbol{X_{i,j}}|\boldsymbol{Z_i}, \boldsymbol{\Theta}) = 1$ when the antibody kinetics model g is deterministic for all values of $\boldsymbol{Z_i}$ and $\boldsymbol{\Theta}$. The two components of the likelihood (the observation process and antibody kinetics model) are therefore combined as $f(\boldsymbol{Y_{i,t}}|\boldsymbol{Z_i}, \boldsymbol{\Theta})$ in the main text. The full system can be represented as a directed acyclic graph (Fig A1).

This problem falls within the remit of binary variable selection: a well described area of research in the context of regression models and a challenging problem as the number of binary variables and resulting model space grows large [1, 2, 3, 4]. Methods such as Stochastic search variable selection and Reversible Jump Markov chain Monte Carlo are well established for model selection tasks, but not sufficient to describe the present problem, where binary outcomes are the result of complex, unobserved epidemiological processes that must be taken into consideration. In particular, we must carefully consider that not all infection events are independent. For example, individuals that are infectious at a given time exert a force of infection on other individuals in the same population, and *a priori* we do not know if an individual experienced many or few infections over their lifetime. Here, we describe a number of priors that can take these processes into account and discuss their implications on inferring individual infection histories and attack rates with the *serosolver* package.

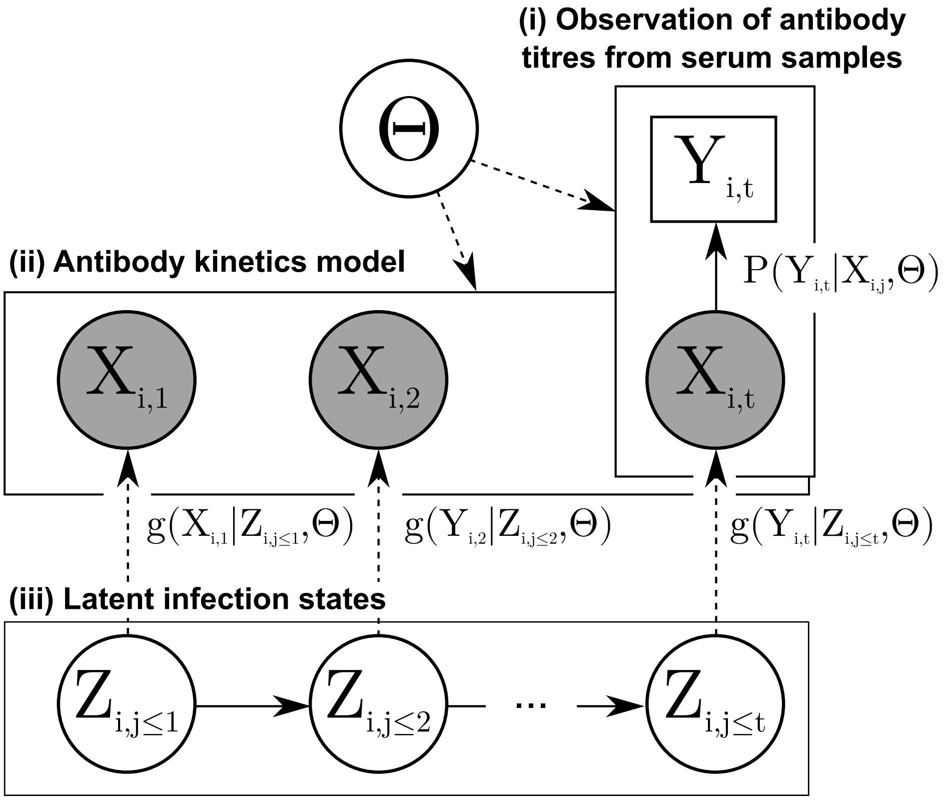

Fig A1. Directed acyclic graph representation of the full model. White circles represent parameters/latent states of interest ($Z_{i,j\leq t}$ shows the infection states with respect to each possible infection time j before observation time t, $\boldsymbol{X_{i,t}}$ shows the set of latent antibody titres at time t (immediately after a possible infection event), and $\boldsymbol{Y_{i,t}}$ shows the set of titre observations at time t). Grey circles represent deterministic latent states, whereas the box around $\boldsymbol{X_{i,t}}$ distinguishes observable from latent states. Solid arrows represent stochastic dependencies, dashed arrows represent deterministic dependencies. The different model levels are shown within boxes.

3 Intuitive prior

Intuitively, an uninformative prior on an indicator random variable, $Z_{i,j}$, might be that $z_{i,j} = 1$ occurs with fixed $p = 0.5$ and $z_{i,j} = 0$ occurs with fixed $q = (1 - p) = 0.5$. More generally:

$$P(Z_{i,j}) = p_{i,j}^{Z_{i,j}}(1 - p_{i,j})^{1-Z_{i,j}} \tag{4}$$

$$P(\boldsymbol{Z_i}) = \prod_{j=1}^{m} p_{i,j}^{Z_{i,j}}(1 - p_{i,j})^{1-Z_{i,j}} \tag{5}$$

$$P(\boldsymbol{Z}) = \prod_{i=1}^{n}\prod_{j=1}^{m} p_{i,j}^{Z_{i,j}}(1 - p_{i,j})^{1-Z_{i,j}} \tag{6}$$

If we consider an individual's infection history $\boldsymbol{Z_i}$ to be a sequence of binary variables, $\boldsymbol{Z_i} = [Z_{i,1}, Z_{i,2}, \ldots, Z_{i,m}]$), then this prior implicitly assumes that the total number of infections experienced by individual i is binomially distributed with mean pm. The total number of infections across all n individuals in a given time period, j, is also binomially distributed with mean pn, where $p = p_{i,j}$ for all individuals and all times. Setting $p_{i,j} = 0.5$

is equivalent to assuming that all infection histories are equally likely: an infection history with all $z_{i,j} = 1$ is as likely as one with all $z_{i,j} = 0$, and as likely as any other individual sequence of 1s and 0s.

Although intuitive, this set of assumptions results in a strong prior on the total number of infections. For example, pm infections are substantially more likely than an infection history with 0 or m total infections. Therefore, in the situation where there is relatively little data, $P(\boldsymbol{Z})$ would bias the inferred infection histories towards pm infections per individual and pn infections per unit time. The posterior probability of an infection history with few infections and large amounts of antibody boosting per infection would be far lower than of an infection history with $0.5m$ infections and low antibody boosting.

In reality, for a disease like influenza, infections likely happen less frequently than every other year, though frequency does vary between individuals. Similarly, although the total number of infections in a given influenza season are well described by a binomial distribution, the distribution of infections across multiple outbreaks is likely over-dispersed relative to the binomial distribution due to between-outbreak variation in severity. A prior that allows us to capture these features would therefore be more desirable.

4 Priors in *serosolver*

There are four options provided in *serosolver* for different infection probability priors. Each option follows a different set of assumptions and definitions, leading to different implications for infection history inference. Table S1 summarises each of these priors and the situations when each one is advised, and the remainder of this section describes their derivations in more detail. The implementation in *serosolver* for each of these priors is described in the Appendix.

4.1 Prior 1, hyper-prior on per-time infection probability

Individuals may be from the same population, which implies that their risks of infection are correlated. Under this prior we assume that individuals are infected by the same infection process during a given time period (i.e. there is a force of infection on the population), and that infection processes are independent across time periods. There is an intuition to this approach which is revealed by the following question: given a sample of n individuals for which we know all n infection states for time j, what is the prior predictive probability that individual $n+1$ was also infected during time j? If we know that 80% of the population was infected at time j, then we should have some prior belief that individual $n+1$ was also infected.

Under this prior, the probability of infection is given as $p = \Phi_j$. Φ is related to the attack rate and therefore gives the probability of any individual in the population becoming infected. Under this prior, the infection generating process is:

$$z_{i,j} \sim \text{Bernoulli}(\phi_j) \tag{7}$$
$$\phi_j \sim h(j) \tag{8}$$

where h is any arbitrary function describing the distribution of Φ. The probability mass function for an individual infection event in discrete time period j is therefore given by:

$$P(Z_{i,j}|\Phi_j = \phi_j) = \phi_j^{Z_{i,j}}(1-\phi_j)^{(1-Z_{i,j})} \tag{9}$$

Thus, the likelihood of observing a particular combination of infections at time j is given by a Bernoulli model:

$$\begin{aligned} P([Z_{1,j}, Z_{2,j}, \ldots, Z_{n,j}]|\Phi_j = \phi_j) &= \textstyle\prod_{i=1}^{n} \phi_j^{Z_{i,j}}(1-\phi_j)^{(1-Z_{i,j})} \\ &= \phi_j^{k_j}(1-\phi_j)^{(n_j-k_j)} \end{aligned}$$

where $k_j = \sum_{i=1}^{n_j}$ is the total number of infections during discrete time period j and n_j is the number of individuals who could be infected during time period j. Retaining the correlation between individuals and adding m infection times to the system, the likelihood of $\boldsymbol{Z}$ conditional on $\boldsymbol{\Phi}$ becomes:

$$P(\boldsymbol{Z}|[\Phi_1 = \phi_1, \Phi_2 = \phi_2, \ldots, \Phi_m = \phi_m]) = \prod_{j=1}^{m} \phi_j^{k_j}(1-\phi_j)^{(n_j-k_j)} \tag{10}$$

where $\boldsymbol{Z}$ is an n by m matrix representing the outcome of m possible infection events for n individuals. The first example in Section 3 above makes the strong assumption that Φ_j is fixed at 0.5 for all discrete times j. To avoid this strong assumption of binomially distributed attack rates, we can assume that all $\boldsymbol{\Phi}$ are unknown parameters to be estimated by defining a prior on $\boldsymbol{\Phi}$. The updated full posterior is given as:

$$P(\boldsymbol{Z}, \boldsymbol{X}, \boldsymbol{\Theta}, \boldsymbol{\Phi}|\boldsymbol{Y}) \propto \prod_{i=1}^{n} \Big(\prod_{t \in t_i} P(\boldsymbol{Y_{i,t}}|\boldsymbol{X_{i,t}}, \boldsymbol{\Theta}) \Big) \prod_{j=1}^{j_{\max}} P(\boldsymbol{X_{i,j}}|\boldsymbol{Z_i}, \boldsymbol{\Theta}) P(Z_{i,j}|\Phi_j) P(\Phi_j) P(\boldsymbol{\Theta}) \tag{11}$$

This structure opens up a number of useful possibilities. For example: $\boldsymbol{\Phi}$ may be defined as a function rather than a variable; different priors may be placed on different discrete times j; $\boldsymbol{\Phi}$ may be inferred explicitly. Fig A2 shows an update of Fig A1 taking into account the infection generating process.

4.1.1 Prior on number of lifetime infections

Assuming that all $P(\boldsymbol{\Phi})$ are independent, the total number of lifetime infections for an individual follows a binomial distribution with $p = \mathbb{E}(\Phi)$. Using a beta prior for $P(\boldsymbol{\Phi})$ with parameters α and β and assuming that α and β are equal for all j, then $P(\sum_j Z_{i,j} = Z_{i,1} + Z_{i,2} + \cdots + Z_{i,m})$ follows a binomial distribution with mean $\frac{\alpha}{\alpha+\beta}$ and $N = m_i$, where m_i is the number of discrete time periods that individual i could be infected in. A binomial prior is intuitive here: if the expectation of the attack rates for all discrete times j is $p = 0.5$, then one would assume *a priori* that individuals are infected in every other time period.

4.2 Prior 2, beta prior on the probability of infection in each time period j

The above prior allows for explicit control over the form of $P(\boldsymbol{\Phi})$, but also results in a large number of additional nuisance parameters that must be estimated (each Φ_j). It is possible to calculate the marginal distribution $P(\boldsymbol{Z})$ under the above prior by integrating out $\boldsymbol{\Phi}$. In terms of MCMC mixing, integrating over possible all possible Φ_j for each j reduces the number of free parameters to be estimated rather than needing to estimate each Φ_j. This is particularly useful because inferring posterior distributions for $\boldsymbol{\Phi}$ and $\boldsymbol{Z}$ simultaneously is practically difficult due to their correlation, particularly when m is large. The reader is referred to related work on the Indian Buffet Process: a stochastic process defining a probability distribution over sparse binary matrices with finite rows and infinite columns [6]. This problem is the related case where binary matrices are not necessarily sparse, and the number of columns can be considered finite. However, there is a potential avenue for infection history inference where the number of infection periods (the number of columns) is not necessarily fixed and finite, and we therefore refer to this work here.

Similar to prior version 1, we define a beta-Bernoulli model for the generation of $\boldsymbol{Z}$ as:

$$z_{i,j} \sim \text{Bernoulli}(\phi_j) \tag{12}$$

$$\phi_j \sim \text{Beta}(\alpha, \beta) \tag{13}$$

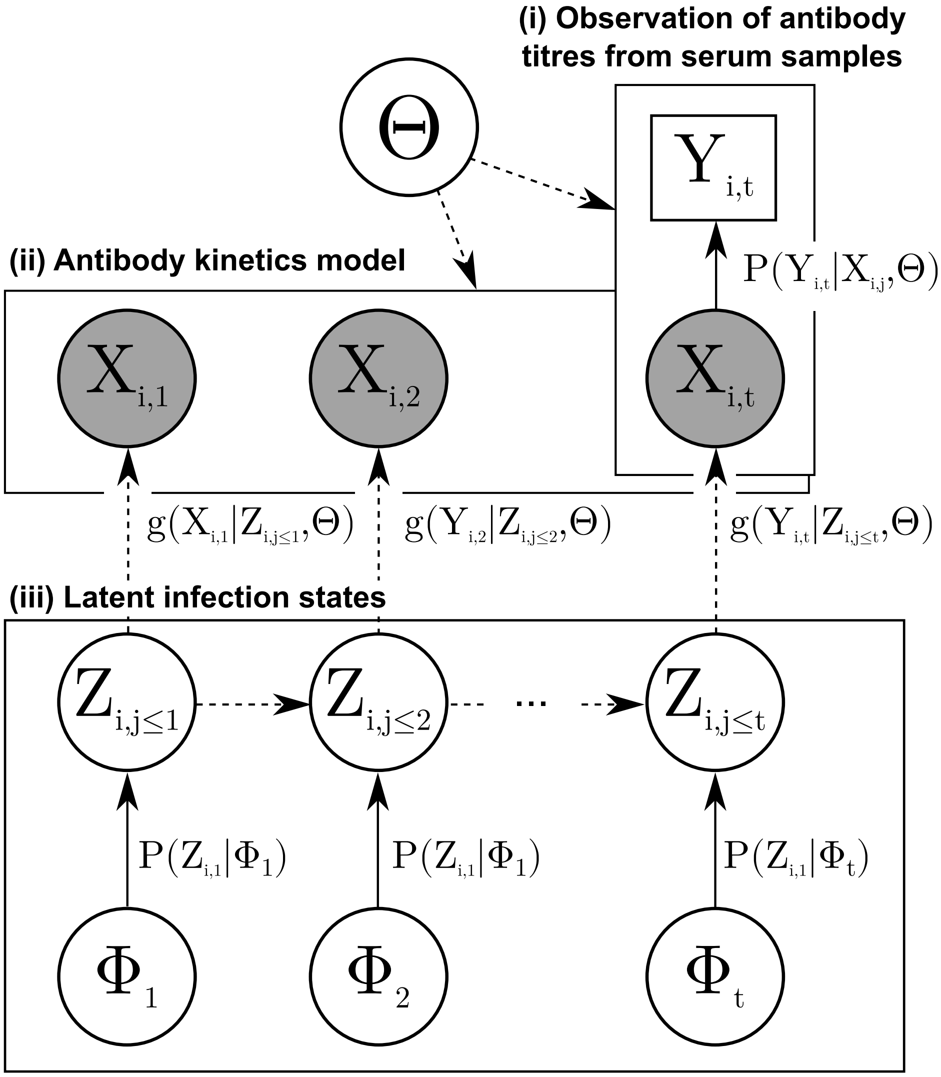

Fig A2. Directed acyclic graph representation of the model with probability of infection term. White circles represent parameters/latent states of interest ($Z_{i,j\leq t}$ shows the infection states with respect to each possible infection time j before observation time t, $\boldsymbol{X_{i,t}}$ shows the set of latent antibody titres at time t (immediately after a possible infection event), and $\boldsymbol{Y_{i,t}}$ shows the set of titre observations at time t). Grey circles represent deterministic latent states, whereas the box around $\boldsymbol{X_{i,t}}$ distinguishes observable from latent states. Φ_t shows the probability of any individual becoming infected during time t. Solid arrows represent stochastic dependencies, dashed arrows represent deterministic dependencies. The different model levels are shown within boxes.

The prior probability of $P(\Phi_j = \phi_j)$ is defined as:

$$P(\Phi_j = \phi_j) = \frac{\phi_j^{\alpha-1}(1-\phi_j)^{\beta-1}}{B(\alpha,\beta)} \tag{14}$$

where $B(\alpha, \beta)$ is the Beta function:

$$B(\alpha, \beta) = \int_0^1 \phi_j^{\alpha-1}(1-\phi_j)^{\beta-1} d\phi_j \tag{15}$$

$$= \frac{\Gamma(\alpha)\Gamma(\beta)}{\Gamma(\alpha+\beta)} \tag{16}$$

$Z_{i,j}$ is independent of all other entries in $\boldsymbol{Z}$, conditional on Φ_j which are also assumed to be independent of all other entries in $\boldsymbol{\Phi}$. $P(Z_{i,j})$ can then be calculated directly without representing $\boldsymbol{\Phi}$ by integrating over all $\boldsymbol{\Phi}$, giving the marginal likelihood of the entire infection history matrix $\boldsymbol{Z}$ as:

$$P(\boldsymbol{Z}) = \prod_{j=1}^{m} \int_0^1 \Big(\prod_{i=1}^{n} P(Z_{i,j} | \Phi_j = \phi_j) \Big) P(\Phi_j = \phi_j) d\phi_j \tag{17}$$

$$= \prod_{j=1}^{m} \frac{B(k_j + \alpha, \beta + n_j - k_j)}{B(\alpha, \beta)} \tag{18}$$

In the MCMC framework, new values for each $Z_{i,j}$ may be sampled directly from this prior as:

$$P(Z_{i,j} = 1 | \boldsymbol{Z_{-i,j}}, \alpha, \beta) = \frac{P(Z_{i,j} = 1, \boldsymbol{Z_{-i,j}})}{P(\boldsymbol{Z_{-i,j}})} \tag{19}$$

$$= \frac{\alpha^{[k_j+1]}\beta^{[n_j-k_j]}}{(\alpha+\beta)^{[n_j+1]}} \frac{(\alpha+\beta)^{[n_j]}}{\alpha^{[k_j]}\beta^{[n_j-k_j]}} \tag{20}$$

$$= \frac{k_j + \alpha}{n_j + \alpha + \beta} \tag{21}$$

Giving the proposal probability of $z_{i,j} = 1$ and $z_{i,j} = 0$ otherwise, where k_j is the number of infections during time j less $z_{i,j}$, and n_j is the number of individuals alive during time j less individual i. The acceptance probability then just becomes the ratio of likelihoods in the Metropolis step.

Values for α and β may be chosen to give a prior on the attack rate with known properties: $\mathbb{E}(k_j) = n\frac{\alpha}{\alpha+\beta}$ and $\mathrm{Var}(k_j) = n\frac{\alpha\beta}{(\alpha+\beta)^2}[1 + (n-1)\frac{1}{\alpha+\beta+1}]$. When $\alpha = \beta$, the attack rate prior has an expectation of $0.5n$, and the variance may be decreased by increasing α and β. For example, values of α and β that have a desired mode and certainty may be chosen by solving the following:

$$\alpha = Mo(c-2) + 1 \tag{22}$$

$$\beta = (1 - Mo)(c-2) + 1 \tag{23}$$

where Mo is the desired mode, and c is analogous to the number of prior observations (i.e. $c = 2$ corresponds to having seen two prior outcomes). Values for α and β that have a particular mean with the largest possible variance are found by solving:

$$\alpha = \bar{\phi}^2 \frac{1 - \bar{\phi}}{\sigma_\phi - \frac{1}{\bar{\phi}}} \tag{24}$$

$$\beta = \alpha \frac{1}{\bar{\phi} - 1} \tag{25}$$

where $\bar{\phi}$ is the desired mean attack rate, and σ_ϕ is the maximum variance for Φ that results in a uni-modal distribution of Φ. Note that values of α and β may be set that lead to a multi-modal distribution of Φ e.g. $\alpha = \beta = \frac{1}{2}$.

4.2.1 Prior on number of lifetime infections

The implicit prior on an individual's number of lifetime infections is the same as in Section 4.1: the beta prior on $P(\mathbf{\Phi})$ with parameters α and β results in a binomial distribution on the total number of lifetime infections with mean $\frac{\alpha}{\alpha+\beta}$ and $N = m_i$, where m_i is the number of discrete time periods that individual i could be infected.

4.3 Prior 3, beta-binomial prior on the total number of lifetime infections

Under this prior, each individual's prior probability of infection is drawn from a Bernoulli distribution with independent success probability, p_i, for all i, but the same p_i for individual i across all discrete times j. This prior captures the idea that individuals may have a tendency to get infected more or less frequently, but the probability of an individual becoming infected is independent of all other individuals. Similar to prior version 2, we model this with a beta-Bernoulli distribution where the probability of infection is a random variable Λ_i. This process is modelled as:

$$
\begin{aligned}
z_{i,j} &\sim \text{Bernoulli}(\lambda_i) \qquad (26)\\
\lambda_i &\sim \text{Beta}(\alpha, \beta) \qquad (27)
\end{aligned}
$$

This places a beta prior on the per-time probability of infection, assuming that all individuals' infection probabilities are independent and not identically distributed (i.e. each individual has a unique Λ_i). The prior probability of a particular infection history for individual i, $\boldsymbol{Z_i}$, is therefore given by a standard beta-Bernoulli distribution with probability Λ_i. It is possible to marginalise over Λ_i to define $P(\boldsymbol{Z_i})$ directly.

If the prior on Λ_i follows the beta distribution (B):

$$
P(\Lambda_i) = \frac{1}{B(\alpha,\beta)} \Lambda_i^{\alpha-1}(1-\Lambda_i)^{\beta-1} \qquad (28)
$$

and the likelihood of $\boldsymbol{Z_i}$ given $\Lambda_i = \lambda_i$ is:

$$
P(\boldsymbol{Z_i}|\Lambda_i = \lambda_i) = \lambda_i^{k_i}(1-\lambda_i)^{m_i-k_i} \qquad (29)
$$

then the marginal distribution of $P(\boldsymbol{Z_i})$ is:

$$
\begin{aligned}
P(\boldsymbol{Z_i}) &= \mathbb{E}_P(P(\boldsymbol{Z_i})|\Lambda_i) \qquad (30)\\
&= \int_0^1 \lambda_i^{k_i}(1-\lambda_i)^{m_i-k_i} P(\lambda_i) d\lambda_i \qquad (31)\\
&= \frac{B(\alpha+k_i, \beta+m_i-k_i)}{B(\alpha,\beta)} \qquad (32)\\
&= \frac{\alpha^{[k_i]}\beta^{[m_i-k_i]}}{(\alpha+\beta)^{[m_i]}} \qquad (33)
\end{aligned}
$$

where $\boldsymbol{Z_i} = [Z_{i,1}, Z_{i,2}, \ldots, Z_{i,m}]$, k_i is the total number of infections experienced by individual i ($\sum_{j=1}^{m} Z_{i,j}$), m_i is the number of discrete time periods that individual i could be infected in, and $r^{[x]}$ denotes the ascending power $r(r+1)\ldots[r+(x-1)]$. The probability mass function for the total number of infections k_i is therefore given by:

$$
P(k_i, m_i|\alpha,\beta) = \binom{m_i}{k_i} \frac{B(\alpha+k_i, \beta+m_i-k_i)}{B(\alpha,\beta)} \qquad (34)
$$

which is the beta-binomial distribution. This prior makes the following assumptions:

1. Each p_i comes from a single draw from the same beta distribution;
2. All Λ_i are equal for a given i (i.e. all $z_{i,j}$ are drawn from the same Bernoulli distribution);
3. All p_j are independent for a given j (i.e. each p_j is drawn from a different distribution for each j).

Formulating the prior in this way allows an explicit prior to be defined through α and β on the total number of infections in a particular infection history $\boldsymbol{Z_i}$, with $\mathbb{E}(k_i) = m\frac{\alpha}{\alpha+\beta}$ and $\mathrm{Var}(k_i) = m\frac{\alpha\beta}{(\alpha+\beta)^2}[1+(m-1)\frac{1}{\alpha+\beta+1}]$. An intuitive uniform prior on an infection history would therefore be that any total number of lifetime infections is equally likely, which is the case where $\alpha = \beta = 1$. In addition, as $\lim \alpha = \beta \to \infty$, $P(k, m|\alpha, \beta) \to \mathrm{Binom}(k, m)$, where any infection history is equally likely. A more informative prior on $\boldsymbol{Z_i}$ is also possible by choosing values for α and β that give a desired mean and variance on the total number of infections per individual.

4.3.1 Prior on attack rates

The assumption of independent individuals and a beta-Bernoulli prior on the total number of lifetime infections places a binomial prior on the attack rate within a given time period j across n individuals. The marginal likelihood of infection in an individual's infection history is the same across all individuals, such that:

$$\begin{aligned}
P(Z_{i,j} = 1|P_{i,j} = p_{i,j}) &= p_{i,j} && (35)\\
P(P_{i,j} = p_{i,j}) &= \frac{p_{i,j}^{\alpha-1}(1-p_{i,j})^{\alpha-1}}{B(\alpha,\beta)} && (36)\\
P(Z_{i,j} = 1) &= \int_0^1 P(Z_{i,j} = 1|P_{i,j} = p_{i,j})P(P_{i,j} = p_{i,j})dp_{i,j} && (37)\\
&= \int_0^1 p_{i,j}\frac{p_{i,j}^{\alpha-1}(1-p_{i,j})^{\alpha-1}}{B(\alpha,\beta)}dp_{i,j} && (38)\\
&= \mathbb{E}(p_{i,j}) && (39)\\
&= \frac{\alpha}{\alpha+\beta} && (40)
\end{aligned}$$

Individuals are independent and therefore all $p_{i,j}$ are independent across j, and α and β are the same for all individuals i. It then follows that $P(\sum \boldsymbol{Z_j} = Z_{1,j} + Z_{2,j} + \cdots + Z_{n,j})$ is binomially distributed with probability $\frac{\alpha}{\alpha+\beta}$ and $N = n$, the number of individuals. Importantly, $P(\sum \boldsymbol{Z_j})$ is binomially distributed with mean $0.5n$ for all $\alpha = \beta$, even in the case where $\alpha = \beta = 1$.

This prior would suggest that the infection status of individual $n + 1$ during time j follows the Bernoulli distribution with $p_j = \frac{\alpha}{\alpha+\beta}$, and the overall number of infections k_j follows the binomial distribution with the same p_j and $N = n_j$. This does fulfil a number of desirable properties: (i) if we know that a proportion $\frac{\alpha}{\alpha+\beta}$ of the population were infected, then the expectation of the attack rate prior would also be $\frac{\alpha}{\alpha+\beta}$; (ii) certainty in the attack rate estimate should increase with increasing n. However, with large n and this binomial prior, the majority of the probability density for the attack rate is in a relatively small region of parameter space, resulting in a prior that strongly influences the posterior, and might therefore swamp the likelihood. Furthermore, given that $\mathbb{E}(p_j) = \frac{\alpha}{\alpha+\beta}$, then necessarily $p_1 = p_2 = \cdots = p_m$ for all m.

4.4 Prior 4, beta prior on the probability of any infection event

The final and perhaps most truly "uninformative" prior comes from the assumption that *all* infections are independent and identically distributed events; belonging to a common group or time period is considered irrelevant and the order does not matter. In this case:

$$z_{i,j} \sim \text{Bernoulli}(\phi) \tag{41}$$
$$\phi \sim \text{Beta}(\alpha, \beta) \tag{42}$$

Such that:

$$P(\Phi = \phi) = \frac{\phi^{\alpha-1}(1-\phi)^{\beta-1}}{B(\alpha,\beta)} \tag{43}$$

$$P(Z_{i,j}|\Phi = \phi) = \phi^{Z_{i,j}}(1-\phi)^{1-Z_{i,j}} \tag{44}$$

and the marginal likelihood of Z is:

$$P(\boldsymbol{Z}) = \int_0^1 \Big(\prod_{j=1}^{m}\prod_{i=1}^{n} P(Z_{i,j}|\Phi = \phi)\Big)P(\Phi = \phi)d\phi \tag{45}$$
$$= \frac{B(k+\alpha, \beta+nm-k)}{B(\alpha,\beta)} \tag{46}$$

where k is the total number of infections across all years and individuals and nm is the total number of possible infection events. This gives the conditional probability that an individual was infected at a given time, and also a distribution to draw new infection states from:

$$P(Z_{i,j} = 1|\boldsymbol{Z_{-i,j}}, \alpha, \beta) = \int_0^1 P(Z_{i,j}|\Phi = \phi)P(\Phi = \phi|\boldsymbol{Z_{-i,j}})d\phi \tag{47}$$
$$= \frac{k_{-i}+\alpha}{nm_{-i}+\alpha+\beta} \tag{48}$$

This assumption has the desirable property of placing a beta prior on both the total number of infections over a lifetime for a given individual and on the total number of infections during a given time. However, these properties are traded off against the strong and potentially unrealistic assumption that infection events are conditionally independent across both time and individuals.

5 Choice of prior

To illustrate the impact of different prior assumptions on infection history and antibody kinetics parameter inference, we ran simulation-recovery experiments in an antigenically variable pathogen system (e.g. influenza) for (i) a beta prior on the per-time probability of infection (Section 4.2), (ii) a beta-binomial prior on total number of lifetime infections (Section 4.3.1) and (iii) a beta prior on the probability of any infection event (Section 4.4) with varying amounts of titre data and beta prior parameters. The simulated sero-survey designs are described in Table A1. For each prior version, we considered three data scenarios: (i) sparse data, only one serum sample and titres against 9 antigenically related (but distinct) viruses taken; (ii) full data, one serum sample and titres against each of the 41 antigenically related viruses (one per year); (iii) additional data, 5 serum samples taken at random intervals between 2000 and 2009, with 41 antigenically related viruses tested for each serum sample. These three data scenarios represent a range of low data contribution to the posterior up to extremely high data contribution. For each data scenario and prior version, we tested 4 beta prior assumptions: (i) neutral prior with $\alpha = \beta = \frac{1}{3}$; (ii) a uniform

prior with $\alpha = \beta = 1$; (iii) weakly informative prior with prior probability of infection mode of 0.15 and high variance, with $\alpha = 1.3$ and $\beta = 2.7$, conceptually similar to a prior informed by 4 previous infection observations; (iv) strongly informative prior with prior probability of infection mode of 0.15 and low variance, with $\alpha = 15.7$ and $\beta = 84.3$, conceptually similar to a prior informed by 100 previous infection observations. We ran the MCMC framework to generate 5 chains of 1000000 iterations for these scenarios with a 200000 iteration burn in period.

**Data set parameters**		**Model parameters**					
n	200	Parameter	Description	Value	Prior lower bound	Prior upper bound	Estimated
Year min	1968	μ_l	Long term antibody boosting	1.8	0	8	Yes
Year max	2009	μ_s	Short term antibody boosting	2.7	0	8	No
m	41	σ_l	Long term cross reactivity	0.1	0	1	Yes
Time resolution (infection states)	annual (48)	σ_s	Short term cross reactivity	0.03	0	1	No
Number of serum samples per individual	1/1/5	τ	Suppression	0.05	0	1	Yes
Number of viruses tested	9/41/41	ω	Waning	0.8	0	1	Yes
Number of titre measurement repeats	1	ϵ	Measurement error	0.8	0	25	Yes
Total number of measurements	Varied	α	Infection history prior	$\frac{1}{3}$/1/1.3/15.7	NA	NA	No
First sample year	2000	β	Infection history prior	$\frac{1}{3}$/1/2.7/84.3	NA	NA	No
Final sample year	2009						
Minimum age (years)	10						
Maximum age (years)	75						
Infection history prior	Priors 2, 3 and 4						

Table A1. Simulation settings to compare different infection history prior assumptions.

Fig A3 shows how placing priors on the per-time probability of infection and the overall probability of infection recovers unbiased estimates of the long-term boosting parameter μ_l for all data and prior scenarios, whereas the prior on per-individual probability of infection is only unbiased with a large amount of data or strong prior information. Under this survey design, using prior versions 2 (beta prior on the per-time probability of infection) or 4 (beta prior on the overall probability of infection) would be recommended for estimating long-term dynamics and attack rates. This is supported by Fig A4, where recovering the true attack rates shows little bias under all scenarios for prior versions 2 and 4, but strong bias in all but the strongest data scenario for prior version 3 (beta-binomial prior on total number of lifetime infections). Attack rate estimation becomes increasingly precise with increasing data availability.

Figs A6–A7 show the ability of these different priors to infer the same individual's infection history using the 'full data' scenario. Prior versions 2 and 4 are able to accurately recover the timing of the individual's infections even under the neutral and uniform priors. Prior version 3 does not recover constrained posterior estimates for the cumulative infection history under all but the strongest prior (Fig A6). However, under the more data rich scenarios, prior version 3 is able to recover unbiased estimates of the true cumulative infection history, despite bias in the inferred attack rates (not shown).

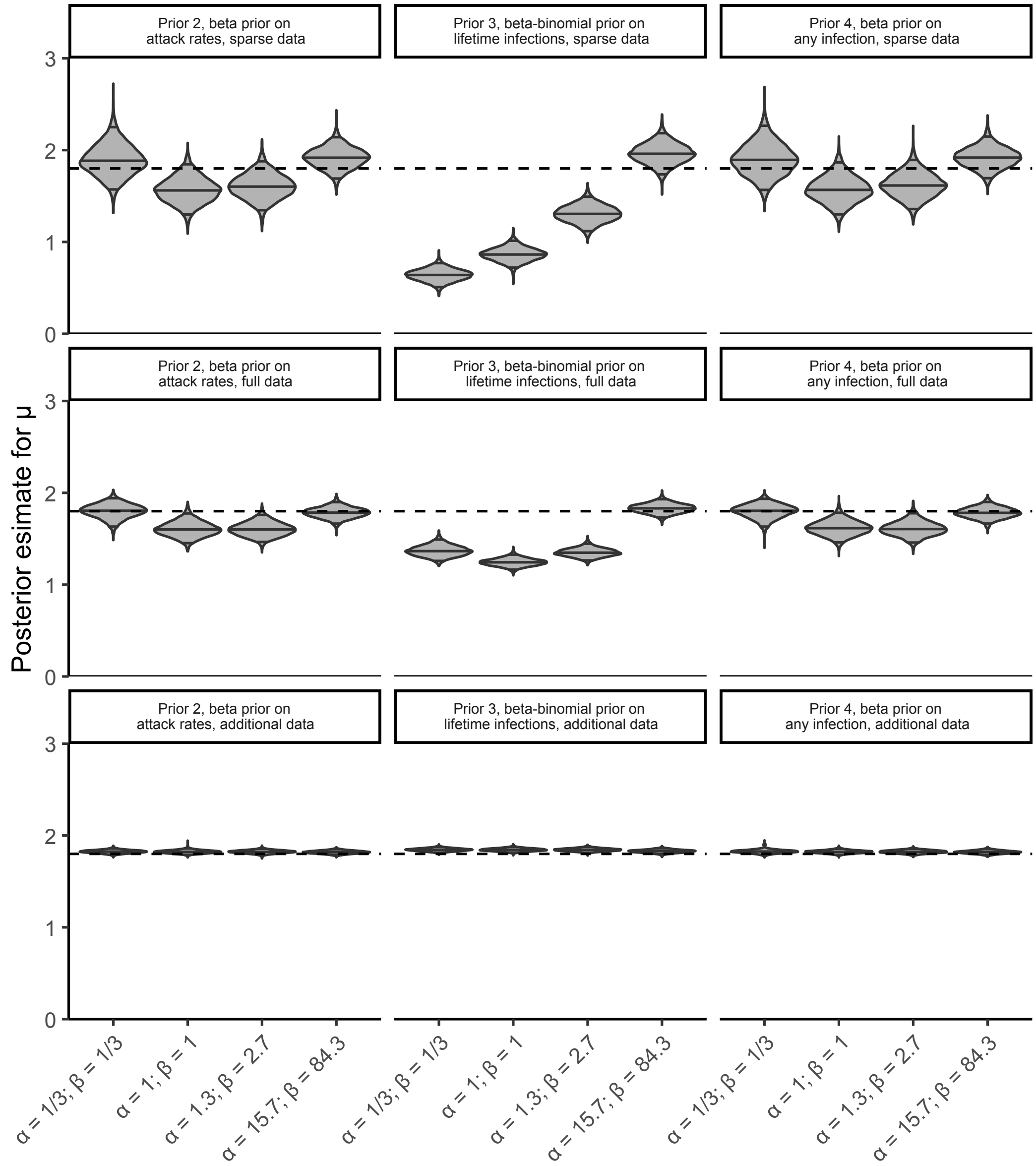

Fig A3. Posterior distribution of long-term boosting parameters μ_l under different prior and data scenarios. Horizontal dashed line shows true parameter value $\mu_l = 1.8$, shaded regions show inferred posterior distribution with medians and 95% credible intervals are shown as solid horizontal lines. X-axis shows assumed beta prior parameters. Left-hand column shows results under prior version 2 (beta prior on the probability of any infection in discrete time period j, Φ_j); middle column shows results under prior version 3 (beta-binomial prior on total number of lifetime infections, Λ_i); right-hand column shows results under prior version 4 (beta prior on probability of any infection event, Φ).

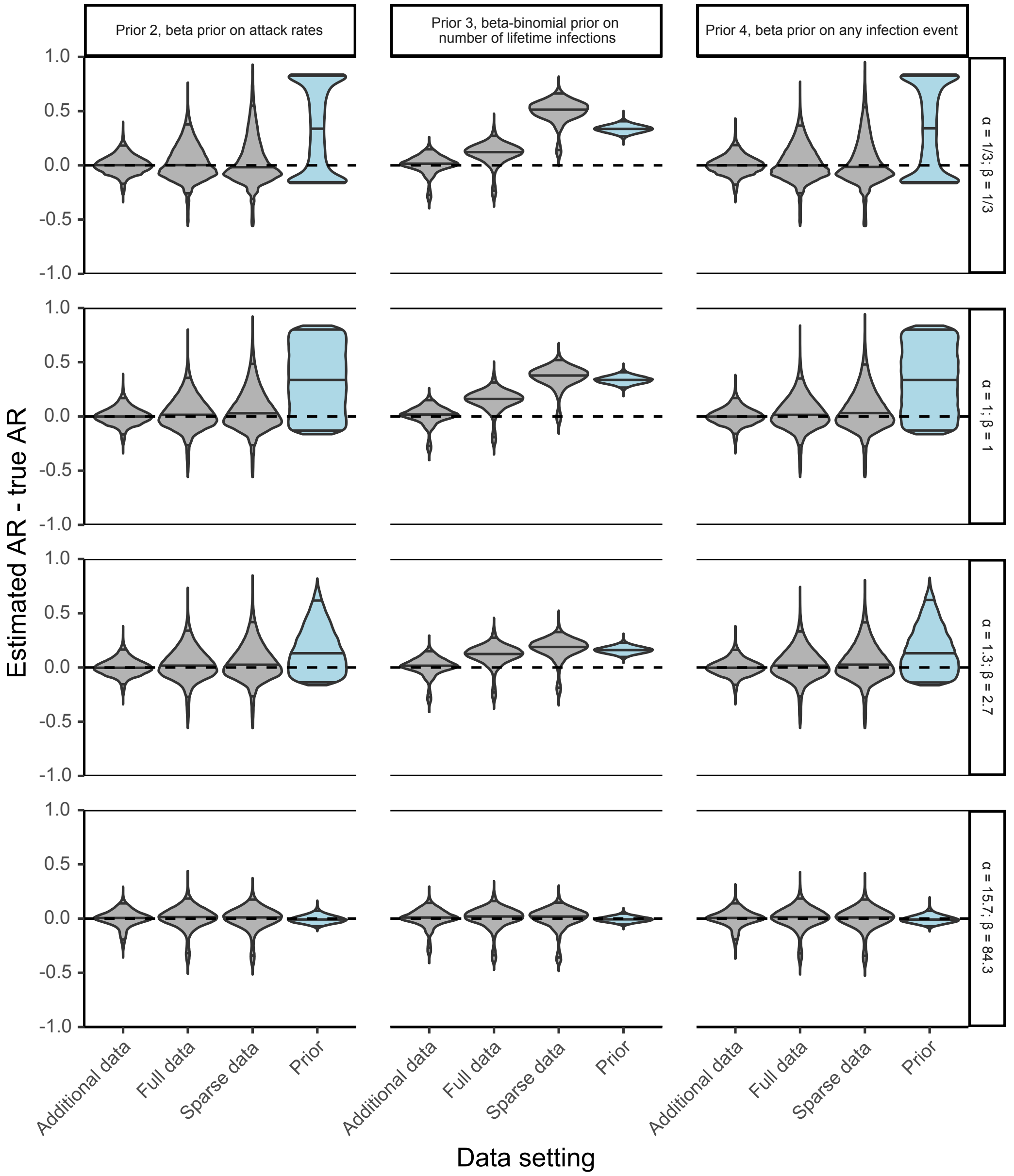

Fig A4. Accuracy of estimated attack rates under the various priors, prior strengths and data scenarios. Violin plots show posterior distribution of inferred attack rate minus the true attack rate. Horizontal lines show posterior medians and 95% credible intervals. Blue violin plot shows attack rate prior - 0.15. X-axis shows decreasing contribution of the data relative to the prior. Top labels show assumed infection history prior version. Right labels show prior parameter settings.

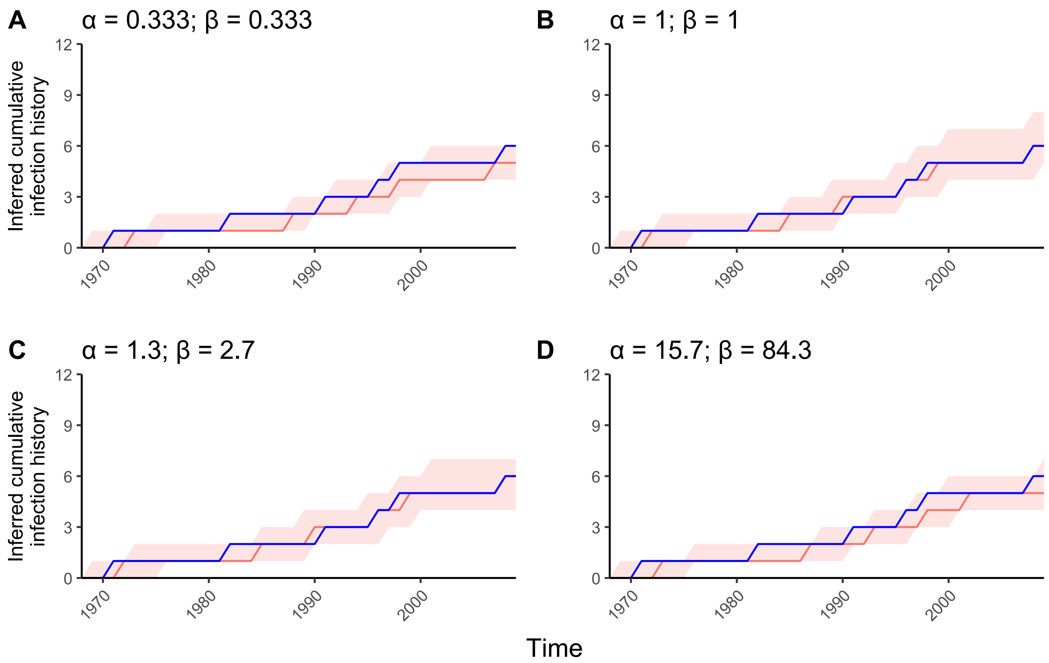

Fig A5. Simulation-recovery of one individual's infection history using prior version 3 (beta prior on attack rates, Φ_j) with various strength priors, full data. Simulation with 200 individuals, 41 viruses tested for each individual, one blood sample taken. Y-axis shows the cumulative number of infections for this individual over time. Red line and shaded region shows posterior median and 95% credible intervals. Blue line shows the true cumulative number of infections over time.

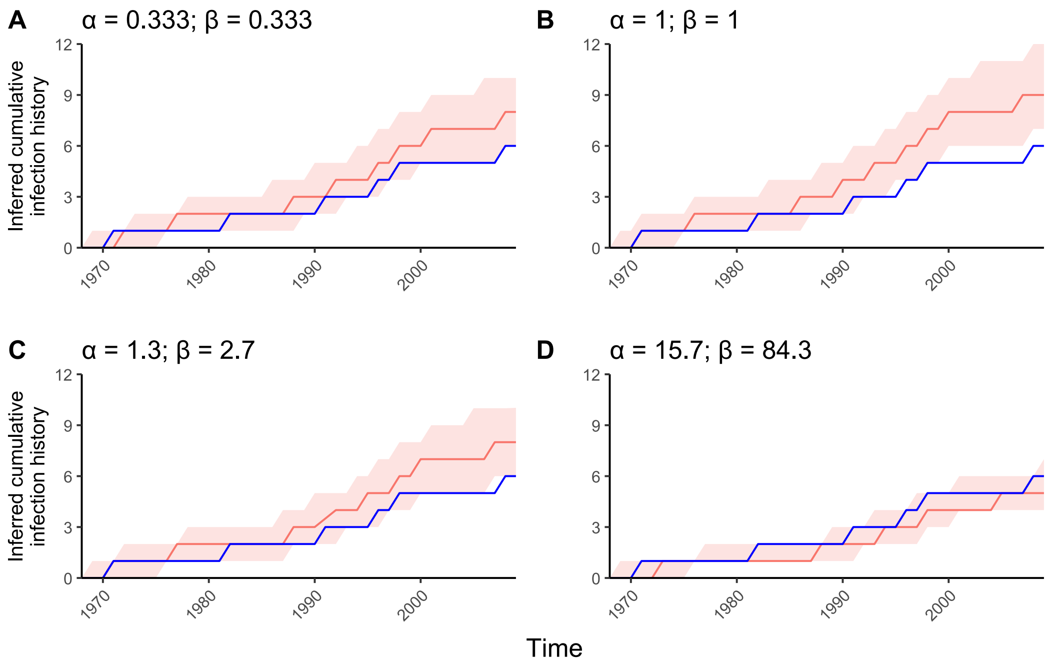

Fig A6. Simulation-recovery of one individual's infection history using prior version 3 (beta-binomial prior on total number of lifetime infections, Λ_i) with various strength priors, full data. Simulation with 200 individuals, 41 viruses tested for each individual, one blood sample taken. Y-axis shows the cumulative number of infections for this individual over time. Red line and shaded region shows posterior median and 95% credible intervals. Blue line shows the true cumulative number of infections over time.

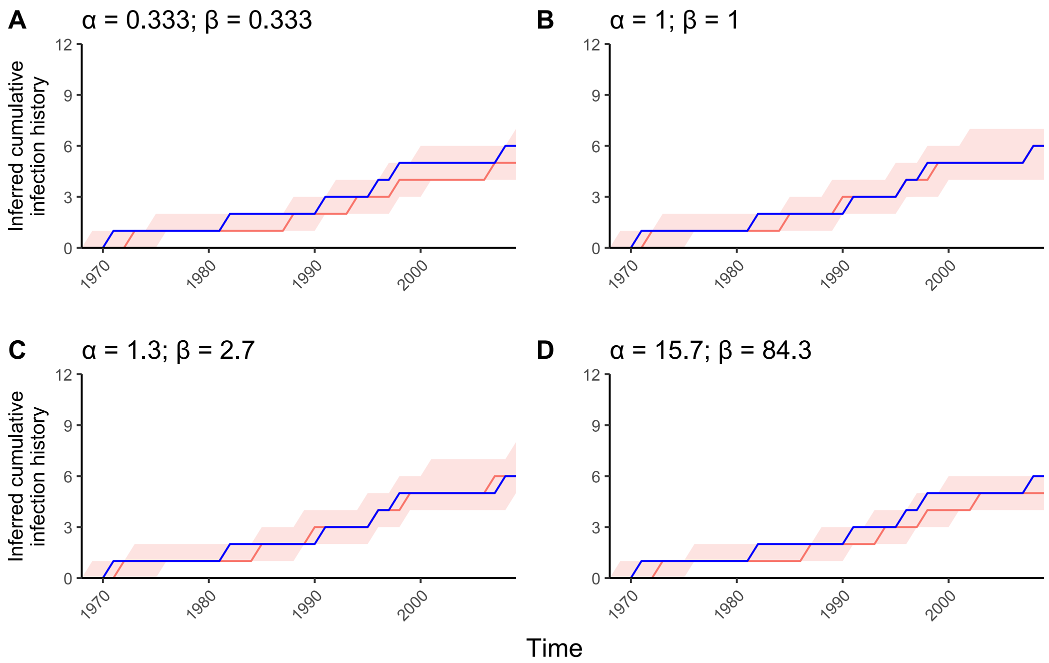

Fig A7. Simulation-recovery of one individual's infection history using prior version 4 (beta prior on any infection event) with various strength priors, full data. Simulation with 200 individuals, 41 viruses tested for each individual, one blood sample taken. Y-axis shows the cumulative number of infections for this individual over time. Red line and shaded region shows posterior median and 95% credible intervals. Blue line shows the true cumulative number of infections over time.

6 Appendix

6.1 Proposal algorithm under prior version 1, hyper-prior on probability of infection

In *serosolver*'s MCMC algorithm, all $\boldsymbol{\Phi}$ are treated as unknown parameters under this prior and therefore sampled alongside $\boldsymbol{\Theta}$. Proposals for $\boldsymbol{Z}$ are made through a random scan across n and m, with a proposed transition of flipping the binary entry for $Z_{i,j}$ i.e. $Z'_{i,j} = 1$ if $Z_{i,j} = 0$, and $Z'_{i,j} = 0$ if $Z_{i,j} = 1$. The sampling algorithm for Z under prior version 1 (Section 4.1) is as follows:

1. With probability *hist_switch_prob*:
   (a) Randomly select a time point j
   (b) Randomly select another time point between 1 and *move_size* time points away from j with uniform probability.
   (c) Randomly select *year_swap_propn**n individuals
   (d) For each of these selected individuals, swap the values of $Z_{i,j}$ and $Z_{i,l}$, adhering to restrictions of birth times (i.e. ignore swap when individuals cannot be infected before they are born)
   (e) Increase/decrease Φ_j and Φ_l proportional to the number of infections gained/lost
   (f) Let $\boldsymbol{Z}$ and $\boldsymbol{\Phi}$ represent the infection history matrix and probability of infection terms before this proposal step, and $\boldsymbol{Z'}$ and $\boldsymbol{\Phi'}$ represent these terms after the proposal step. Set $\boldsymbol{Z} = \boldsymbol{Z'}$ and $\boldsymbol{\Phi} = \boldsymbol{\Phi'}$ with the following acceptance ratio:

$$A((\boldsymbol{Z'}, \boldsymbol{\Phi'}), (\boldsymbol{Z}, \boldsymbol{\Phi})) = \min(1, \frac{P(\boldsymbol{Z'}, \boldsymbol{\Theta}, \boldsymbol{\Phi'}|\boldsymbol{Y})}{P(\boldsymbol{Z}, \boldsymbol{\Theta}, \boldsymbol{\Phi}|\boldsymbol{Y})}) \quad (49)$$

2. Otherwise:
   (a) Select a random proportion *hist_sample_prob* of n individuals
   (b) For each individual, i, propose a new infection history $\boldsymbol{Z'_i}$ by performing one of the following steps:
      i. Perform a "flip" step with probability *1 - swap_propn*:
         A. Select *inf_propn**m_i time points, where m_i is the number of time points that individual i could be infected
         B. Perform a binary flip on each of these times, $Z'_{i,j} = 1 - Z_{i,j}$
      ii. Otherwise, perform a "swap" step:
         A. Randomly select a location j
         B. Select a random location, l, 0 to *move_size* time steps away with equal probability
         C. Set $Z_{i,l} = Z_{i,j}$ and $Z_{i,j} = Z_{i,l}$
   (c) For each sampled individual, independently accept or reject the proposed new infection state with the acceptance ratio:

$$A((\boldsymbol{Z'_i}, \boldsymbol{\Phi'}), (\boldsymbol{Z_i}, \boldsymbol{\Phi})) = \min(1, \frac{P(\boldsymbol{Z'_i}, \boldsymbol{\Theta}, \boldsymbol{\Phi}|\boldsymbol{Y_i})}{P(\boldsymbol{Z_i}, \boldsymbol{\Theta}, \boldsymbol{\Phi}|\boldsymbol{Y})}) \quad (50)$$

If *hist_opt* is set to 1 by the user, then step 2 above is automatically tuned, whereby *hist_sample_prob* is increased or decreased to achieve a desired acceptance rate (usually between 0.25 and 0.4). It is also possible to manually tune *move_size*, *swap_propn*, *year_swap_propn*

and *hist_switch_prob* to improve the acceptance rate of the other proposal steps, though *serosolver* does not currently do this automatically. The acceptance rate of steps 1 and 2 above are printed at regular intervals during the MCMC procedure, which the user may use to tweak these inputs. Further automated tuning remains a direction for further development of the package.

6.2 Proposal algorithm under prior version 2, beta prior on attack rates

The proposal algorithm for prior version 2 is similar to that of prior version 1, but rather than performing a "flip" step, infection history entries are proposed in a Gibbs-like fashion conditional on the infection status of all other individuals at that time point.

1. With probability *hist_switch_prob*:
   (a) Randomly select a time point j
   (b) Select another time point between 1 and *move_size* time points away from j with uniform probability, where $j \neq l$
   (c) Randomly select *year_swap_propn**n individuals and filter for individuals that were alive during both time points
   (d) For each of these selected individuals, swap the values of $Z_{i,j}$ and $Z_{i,l}$
   (e) Let $\boldsymbol{Z}$ represent the infection history matrix before this proposal step, and $\boldsymbol{Z'}$ represent it after. Set $\boldsymbol{Z} = \boldsymbol{Z'}$ with the following acceptance ratio:

$$A(\boldsymbol{Z'}, \boldsymbol{Z}) = \min(1, \frac{\prod_i f(\mathbf{Y_i}|\boldsymbol{Z'_i}, \boldsymbol{\Theta})P(\boldsymbol{Z'_i})}{\prod_i f(\mathbf{Y_i}|\boldsymbol{Z_i}, \boldsymbol{\Theta})P(\boldsymbol{Z_i})}) \tag{51}$$

2. Otherwise:
   (a) Select a random proportion *hist_sample_prob* of n individuals
   (b) For each individual, i, propose a new infection history $\boldsymbol{Z'_i}$ by performing one of the following steps:
      i. Sample new values for $\boldsymbol{Z_i}$ with probability *1 - swap_propn* as follows:
         A. Select *inf_propn**m_i time points, where m_i is the number of time points that individual i could be infected. For each time point, j:
         B. Calculate the number of infected individuals less the selected individual $k_j = (\sum_x Z_{x,j}) - Z_{i,j}$
         C. Calculate the number of individuals that could be infected during time j, n_j
         D. Set $Z_{i,j} = 1$ with probability $\frac{k_j+\alpha}{n_j+\alpha+\beta}$, and $Z_{i,j} = 0$ otherwise
         E. Accept the proposed move with the acceptance ratio, noting that by sampling directly from the prior $P(\boldsymbol{Z})$ that this cancels out in the Metropolis ratio:

$$A(\boldsymbol{Z'_i}, \boldsymbol{Z_i}) = \min(1, \frac{f(\mathbf{Y_i}|\boldsymbol{Z'_i}, \boldsymbol{\Theta})}{f(\mathbf{Y_i}|\boldsymbol{Z_i}, \boldsymbol{\Theta})}) \tag{52}$$

      ii. Otherwise, perform a "swap" step:
         A. Select a location j
         B. Select a location, l, 0 to *move_size* time steps away with equal probability
         C. If $Z_{i,l} \neq Z_{i,j}$, set $Z_{i,l} = Z_{i,j}$ and $Z_{i,j} = Z_{i,l}$ with the acceptance ratio:

$$A(\boldsymbol{Z'_i}, \boldsymbol{Z_i}) = \min(1, \frac{f(\mathbf{Y_i}|\boldsymbol{Z'_i}, \boldsymbol{\Theta})P(\boldsymbol{Z'_i})}{f(\mathbf{Y_i}|\boldsymbol{Z_i}, \boldsymbol{\Theta})P(\boldsymbol{Z_i})}) \tag{53}$$

As in prior version 1, if *hist_opt* is set to 1 by the user then *hist_sample_prob* is tuned to achieve a user-specified acceptance rate. It is also possible to manually tune *move_size* and *swap_propn*.

6.3 Proposal algorithm under prior version 3, beta prior on per-individual infection probability

In practice, assuming that all j are exchangeable, it is possible to sample $Z_{i,j}$ from the prior directly in a Gibbs-like fashion, which leads to far more efficient proposals. Rather than either moving to a proposed location or staying in the previous location, we can think about the proposal steps as offering the algorithm two choices, to either add or remove and infection:

1. For an individual i, choose a random location, j, from the infection history vector, $\boldsymbol{Z_i}$
2. Remove element j to give $\boldsymbol{Z_{i,-j}}$
3. There are now two potential moves to get back to a vector with the same dimensions as $\boldsymbol{Z_i}$. Set $Z_{i,j} = 1$ or $Z_{i,j} = 0$.

Let $\boldsymbol{Z_i'}$ be the case where $Z_{i,j} = 0$ and $\boldsymbol{Z_i}$ be the case where $Z_{i,j} = 1$. More generally, the proposals can be drawn from:

$$P(\text{propose } \boldsymbol{Z_i}) = \frac{g(\boldsymbol{Z_i}|\boldsymbol{Z_{i,-j}})}{g(\boldsymbol{Z_i}|\boldsymbol{Z_{i,-j}}) + g(\boldsymbol{Z_i'}|\boldsymbol{Z_{i,-j}})} \tag{54}$$

In the case of the binomial prior on k (where $P(Z_{i,j} = 1) = 0.5$ when $\alpha = \beta = \infty$), we would have a proposal such that $g(\boldsymbol{Z_i}|\boldsymbol{Z_i'}) = g(\boldsymbol{Z_i'}|\boldsymbol{Z_i}) = g(\boldsymbol{Z_i'}|\boldsymbol{Z_i'}) = g(\boldsymbol{Z_i}|\boldsymbol{Z_i})$. In this case, the probability of proposing $\boldsymbol{Z_i'}$ is the same as the probability of proposing $\boldsymbol{Z_i}$ (i.e. 50/50). However, if we explicitly define $g(\boldsymbol{Z_i}|\boldsymbol{Z_{i,-j}})$ and $g(\boldsymbol{Z_i'}|\boldsymbol{Z_{i,-j}})$ then we can control the proposal distribution and therefore sample from the prior:

$$\begin{aligned} g(\boldsymbol{Z_i}|\boldsymbol{Z_{i,-j}}, \alpha, \beta) &= f(Z_{i,j} = 1|\boldsymbol{Z_{i,-j}}, \alpha, \beta) && (55) \\ g(\boldsymbol{Z_i'}|\boldsymbol{Z_{i,-j}}, \alpha, \beta) &= f(Z_{i,j} = 0|\boldsymbol{Z_{i,-j}}, \alpha, \beta) && (56) \end{aligned}$$

$$\begin{aligned} f(Z_{i,j} = 1|\boldsymbol{Z_{i,-j}}, \alpha, \beta) &= \frac{P(\boldsymbol{Z_i})}{P(\boldsymbol{Z_{i,-j}})} && (57) \\ &= \frac{\alpha^{[k+1]}\beta^{[m-k]}}{(\alpha+\beta)^{[m+1]}} \frac{(\alpha+\beta)^{[m]}}{\alpha^{[k]}\beta^{[m-k]}} && (58) \\ &= \frac{\alpha + k}{\alpha + \beta + m - 1} && (59) \end{aligned}$$

$$\begin{aligned} f(Z_{i,j} = 0|\boldsymbol{Z_{i,-j}}) &= 1 - f(Z_{i,j} = 1|\boldsymbol{Z_{i,-j}}, \alpha, \beta) && (60) \\ &= \frac{\beta + m - k - 1}{\alpha + \beta + m - 1} && (61) \end{aligned}$$

where $k = \sum \boldsymbol{Z_{i,-j}}$, and α and β are the left and right parameters of the beta distribution. Following this proposal (which is equivalent to sampling from the prior for $Z_{i,j}$, the proposal is accepted based on the Metropolis acceptance probability:

$$A(\boldsymbol{Z_{new}}, \boldsymbol{Z_{old}}) = \min(1, \frac{P(\boldsymbol{Y}|\boldsymbol{Z_{new}}, \boldsymbol{\Theta})}{P(\boldsymbol{Y}|\boldsymbol{Z_{old}}, \boldsymbol{\Theta})}) \tag{62}$$

6.4 Proposal algorithm under prior version 4, beta prior on overall probability of infection

The proposal algorithm for prior version 4 is identical to prior version 2, with only three changes:

1. In step 2(b)(i)B, k_j is replaced with $k_{-ij} = (\sum_x \sum_y Z_{x,y}) - Z_{i,j}$
2. In step 2(b)(i)C, n_j is replaced by nm
3. $P(\boldsymbol{Z})$ is the prior as described in Section 4.4

References

[1] Lamnisos, Demetris, Griffin, Jim E. and Steel, Mark F.J. Adaptive Monte Carlo for Bayesian Variable Selection in Regression Models. Journal of Computational and Graphical Statistics. 2013;22(3):729-748 doi:10.1080/10618600.2012.694756

[2] George, Edward I. and McCulloch, Robert E. Approaches for Bayesian Variable Selection. Statistica Sinica. 1997;7:339-373 doi:10.2307/24306083

[3] O'Hara, R. B. and Sillanpää, M. J. A review of Bayesian variable selection methods: what, how and which. Bayesian Analysis. 2009;4(1):85-117 doi:10.1214/09-BA403

[4] He, Qianchuan and Lin, Dan-Yu. A Variable Selection Method for Genome-wide Association Studies. Bioinformatics. 2011;27(1):1-8 doi:10.1093/bioinformatics/btq600

[5] Kerman, Jouni Neutral noninformative and informative conjugate beta and gamma prior distributions. Electronic Journal of Statistics. 2011;5:1450-1470 doi:10.1214/11-EJS648

[6] Griffiths, Thomas L. and Ghahramani, Zoubin The Indian Buffet Process: An Introduction and Review. Journal of Machine Learning Research. 2011;12:1185-1224 doi:10.5555/1953048.2021039

[7] Butler, Ken and Stephens, Michael A. The Distribution of a Sum of Independent Binomial Random Variables. Methodol Comput Appl Probab. 2017;19:557–571 doi:10.1007/s11009-016-9533-4

[8] Liu, Boxiang and Quartermous, Thomas Approximating the Sum of Independent Non-Identical Binomial Random Variables. The R Journal Vol. 10/1, July 2018 ISSN 2073-4859
