# Supplementary material for: An open source tool to infer epidemiological and immunological dynamics from serological data: serosolver
Source: PLoS Comput Biol. 2020 May 4;16(5):e1007840. doi: 10.1371/journal.pcbi.1007840 (PMC7241836; doi:10.1371/journal.pcbi.1007840)
Supplement: S2 Text — (PDF) [file pcbi.1007840.s002.pdf]

An open source tool to infer epidemiological and immunological dynamics from serological data: serosolver

Supporting Text 2: additional antibody kinetics and code update guide

Additional immunological mechanisms included in serosolver

Non-linear waning

As an alternative to the persistent long term and transient short term model described above, *serosolver* includes an option to have non-linear waning of the antibody response. Here, the long term boost (μ_1) and the long-term cross-reactive response (σ_1) are both set to 0 and the single parameter that describes waning ω is replaced with the piece wise linear function,

$$\omega = \omega_1 t_m + \mathbb{1}_{(t_m > t_{change})} \omega_2 t_m \tag{1}$$

where $t_m = k - m$ is the time since infection with strain m, $\omega_2 = -\omega_1 \kappa$ and $\kappa \in (0, 1)$. The parameter t_{change} indicates the time at which the slope of the antibody response changes following infection.

Additional boosting assumptions

There are optional mechanisms included in *serosolver* that account for titre-dependent and strain-dependent boosting. The strain-specific antibody boosting mechanism is included to allow for different levels of boosting for distinct strains or between clusters.

In place of a global long-term boosting parameter, μ_1, individual strain level or group level long-term boosting parameters can be passed into the model such that μ_1 is replaced by μ_c, where c is the index of the boosting parameter for the infecting strain. The hierarchical structure of μ_c is flexible: each infecting strain may have a unique, independent μ_c; different infection strains may have shared or separate μ_c, for example within a single antigenic cluster; all μ_c may be estimated independently or drawn from a common distribution. In the latter case, the following term is added to the model:

$$\mu_c \sim \mathcal{N}(\bar{\mu_l},\, \sigma^2_{\mu_l}) \tag{2}$$

where $\bar{\mu_l}$ is the mean long term boost, σ_{μ_l} is the standard deviation, both parameters to be estimated or fixed.

The titre-dependent boosting mechanism accounts for potential antibody ceiling effects, whereby antibody boosting is decreased at higher initial titres [1,2]. For each infection, m, the predicted log antibody titre in Equation 2 in the main text is multiplied by $b(m)$,

$$b(m) = \begin{cases} \max\{0, 1 - \gamma x_{im}^{m}\} & \text{if} \quad x_{im}^{m} \leq x_{change} \; ; \\ \max\{0, 1 - \gamma x_{change}\} & \text{else} \end{cases} \tag{3}$$

where γ is a fitted parameter and x_{change} is the titre-dependent boosting threshold. Initial titres above x_{change} do not experience any further boosting suppression.

Modifying serosolver's code to incorporate additional antibody kinetics models

Modifying source code can be challenging; however, it is our hope that *serosolver* will be extended and branched to test novel biological hypotheses. We have therefore structured the code with the hope that new mechanisms and assumptions can be added without major structural changes to the code base for a user with intermediate R and C++ programming experience. Table B1 outlines the locations in the source code that must be changed to modify the model called by `create_posterior_func`.

All of the plotting code, simulation code (eg. `simulate_data`), and `create_posterior_func`, ultimately calls `titre_data_fast` (L27 of `src/infection_model_fast.cpp`). Making the changes in Table B1 will therefore also update any post-processing, simulation code and the posterior function. Users should include additional model parameters in the parameter vector `theta`. For example, in `example_par_tab`, the long-term boosting parameter "mu" is described. This parameter is automatically located and then extracted at L65 of `infection_model_fast.cpp`. Note that the cross-reactivity matrices for long and short term antibody boosting, `antigenic_map_long` and `antigenic_map_short` are pre-calculated using `create_cross_reactivity_vector`. For example, at L178 and L342 in `simulate_data.R`, and L404 and L503 in `posteriors.R`. The rest of the arguments to `titre_data_fast` pass the current infection history matrix and vectors to control the titre data indexing.

Changes need to be made to `titre_data_fast` in 3 places: (i) extracting model parameters from L65; (ii) selecting the model version to use at L100; (iii) choosing the correct model to solve based on the options using an `ifelse` statement from L124. For computational speed reasons, the model solving code of `titre_data_fast` is directly integrated into the infection history proposal function for prior versions 2 and 4, `inf_hist_prop_prior_v2_and_v4` at L163 of `proposal.cpp`. This code must therefore also be changed in the same way as for `titre_data_fast`. The equivalent 3 locations in `proposal.cpp` are: (i) L296; (ii) L331; (iii) L536.

References

1. Jacobson RM, Grill DE, Oberg AL, Tosh PK, Ovsyannikova IG, Poland GA. Profiles of influenza A/H1N1 vaccine response using hemagglutination-inhibition titers. Human Vaccines & Immunotherapeutics. 2015;11(4):961–969. doi:10.1080/21645515.2015.1011990.

2. Petrie JG, Ohmit SE, Johnson E, Cross RT, Monto AS. Efficacy studies of influenza vaccines: effect of end points used and characteristics of vaccine failures. J Infect Dis. 2011;203(9):1309–1315. doi:10.1093/infdis/jir015.

Table B1. Summary of functions and files that must be changed to incorporate new antibody kinetics parameters/models.

Purpose	Object/function	File	Note
Add new row to input parameter data frame for any new model parameters. Model parameters should be given (i) a name; (ii) a value; (iii) whether the parameter is fixed (1) or estimated (0); (iv) lower bound; (v) upper bound; (vi) lower bound for random starting value; (vii) upper bound for random starting value; (viii) parameter type (1 for kinetics parameter, 0 for a model option flag)	`par_tab`	Input object, specified in a file called `par_tab.csv`	NA
Add header for new antibody kinetics functions	NA	`src/boosting_functions_fast.h`	New functions added to `src/boosting_functions_fast.cpp` must first be defined here
Add new antibody kinetics model code	NA	`src/boosting_functions_fast.cpp`	Note that arguments to this function should take exactly the parameters needed for the model. The other function arguments (infection times, indices etc) should be taken from `titre_data_fast_individual_base`
Add calls to new antibody kinetics code	`titre_data_fast` and `inf_hist_prop_prior_v2_and_v4`	`src/infection_model_fast.cpp` and `src/proposal.cpp`	Setup for the function calls should be added to L65 and L100 for `titre_data_fast`, and L296 and L331 for `inf_hist_prop_prior_v2_and_v4`
