# Supplementary material for: An open source tool to infer epidemiological and immunological dynamics from serological data: serosolver
Source: PLoS Comput Biol. 2020 May 4;16(5):e1007840. doi: 10.1371/journal.pcbi.1007840 (PMC7241836; doi:10.1371/journal.pcbi.1007840)
Supplement: S3 Text — (HTML) [file pcbi.1007840.s003.html]

Paper case study 1: serosolver


# Paper case study 1: serosolver

# Overview

This vignette provides all of the analyses for case study 1 in the accompanying package and paper. Briefly, the first case study aims to 1) reconstruct the unobserved infection dynamics from measured titres collected several months apart, 2) examine these infection dynamics stratified by available demographic variables, such as vaccination status and age, and 3) estimate biological parameters shaping the short-term antibody response. All of the functions used here are well documented and have many tunable arguments, and we therefore encourage users to refer to the helps files.

This vignette demonstrates only how to reproduce the MCMC chains, simulate data, assess model fits and assess chain convergence. Code to reproduce figures from the main text in the accompanying paper can be found in the `inst/extdata/scripts` folder of the package.

# Setup

## Installation and requirements

`serosolver` may be installed from github using the `devtools` package. There are a number of additional packages that we need for this analysis.

```
# Required to run serosolver
devtools::install_github("seroanalytics/serosolver")
library(serosolver)
library(plyr)
library(data.table)

## Required for this analysis
library(reshape2)
library(foreach)
library(doParallel)
library(bayesplot)
library(coda)
library(ggplot2)
library(viridis)
library(ggpubr)

# set up cluster
set.seed(0)
cl <- makeCluster(5)

## Note that this vignette was generated on a Windows machine,
## and the setup for parallelisation is different on a Linux or Mac:

if(Sys.info()[["sysname"]]=="Darwin" | Sys.info()[["sysname"]]=="Linux"){
  library(doMC)
  library(doRNG)
  registerDoMC(cores=5)
}else{
  registerDoParallel(cl)
}
```

## Assumptions

In this analysis, serological samples were taken between 2009 and 2012 and therefore all time variables are relative to this time period. We are interested in inferring infections and attack rates at a quarterly resolution, and therefore set `resolution` to 4. Our primary outcome of interest is to infer unbiased attack rates, and we therefore use the version of the code with a beta prior on per-time attack rates, `prior_version=2`. We set these parameters at the start of the analysis. Additionally, we assume that the samples are tested against the same virus.

```
filename <- "case_study_1"
resolution <- 4 ## set to 4 for quarterly resolution
sample_years <- 2009:2012

serosolver::describe_priors()
#> Which version to use in run_MCMC? The following text describes the proposal step for updating infection histories.
#> Version 1: Beta prior on per time attack rates. Explicit FOI on each epoch using probability of infection term. Proposal performs N `flip` proposals at random locations in an individual's infection history, switching 1->0 or 0->1. Otherwise, swaps the contents of two random locations
#> Version 2: Beta prior on per time attack rates. Gibbs sampling of infection histories as in Indian Buffet Process papers, integrating out each probability of infection term.
#> Version 3: Beta prior on probability of infection for an individual, assuming independence between individuals. Samples from a beta binomial with alpha and beta specified by the par_tab input. Proposes nInfs moves at a time for add/remove, or when swapping, swaps locations up to moveSize time steps away
#> Version 4: Beta prior on probability of any infection. Gibbs sampling of infection histories using total number of infections across all times and all individuals as the prior
prior_version <- 2
```

## Preparing the data

The data used in this analysis are haemagglutination inhibition (HI) titres against A/H1N1pdm09 that began circulating in 2009. The raw data have been pre-processed to both convert them into a form usable for `serosolver` and to separate the data into vaccinated and unvaccinated data sets. Given that this analysis uses titres from a single virus it is not necessary to define an antigenic map.

```
## Read in titre data
# unvaccinated
input_dat_path <- system.file("extdata", "HKdata_h1n1_unvac.csv", package = "serosolver")
input_dat <- read.csv(file = input_dat_path, header = TRUE)
# vaccinated
# input_dat_path2 <- system.file("extdata", "HKdata_h1n1_vac.csv", package = "serosolver")
# input_dat_vac <- read.csv(file = input_dat_path2, header = TRUE)

indivs <- unique(input_dat$individual) #all individuals

# Subset data for indivs
titre_dat <- input_dat[input_dat$individual %in% indivs,
                       c("individual","virus","titre","samples","DOB")]
titre_dat$individual <- match(titre_dat$individual, indivs)

titre_dat <- unique(titre_dat)
titre_dat <- plyr::ddply(titre_dat,.(individual,virus,samples),
                         function(x) cbind(x,"run"=1:nrow(x),"group"=1))
print(head(titre_dat))
#>   individual virus titre samples  DOB run group
#> 1          1  8036     0    8039 8036   1     1
#> 2          1  8036     0    8040 8036   1     1
#> 3          1  8036     7    8044 8036   1     1
#> 4          1  8036     7    8047 8036   1     1
#> 5          2  8036     0    8039 8036   1     1
#> 6          2  8036     5    8041 8036   1     1

strain_isolation_times <- seq(sample_years[1]*resolution+1, sample_years[4]*resolution, by=1)
```

NOTE: vaccinated and unvaccinated data are run separately in this vignette.

Finally, we must specify the `par_tab` data frame, which controls which parameters are included in the model, which are fixed, and their uniform prior ranges. Given that we are integrating out the probability of infection terms under prior version 2, we must remove these parameters from `par_tab`. Furthermore, we remove parameters relating to cross-reactivity (as we are interested in only a single-strain) and antigenic seniority (as the timescale is short). We set alpha and beta to 1/3 to define the beta prior on attack rates.

```
par_tab_path <- system.file("extdata", "par_tab_base.csv", package = "serosolver")
par_tab <- read.csv(par_tab_path, stringsAsFactors=FALSE)

## Set parameters for beta and alpha to 1
par_tab[par_tab$names %in% c("alpha","beta"),"values"] <- c(1/3,1/3)
## Maximum recordable log titre in these data is 9
par_tab[par_tab$names == "MAX_TITRE","values"] <- 9

## Remove phi parameters, as these are integrated out under prior version 2
par_tab <- par_tab[par_tab$names != "phi",]

## Fix cross reactivity and antigenic seniority
par_tab[par_tab$names %in% c("tau","sigma1","sigma2"),"fixed"] <- 1 
## mu, tau, sigma1, and sigma2 are fixed
par_tab[par_tab$names %in% c("tau","sigma1","sigma2"),"values"] <- 0 
## set these values to 0
```

## Summary

- Choose resolution, attack rate priors and reference time for “the present”
- Read in titre data and convert into a useable form for `serosolver`
- Generate antigenic map for the exposing virus (if needed)
- Generate parameter control table for MCMC

# Running the MCMC

We are now ready to fit our model. We will fit multiple chains in parallel, though the below analysis could easily be replicated by running chains sequentially. Starting conditions for the MCMC chain must be generated that return a finite likelihood. The user may modify many of the MCMC control parameters, though the defaults are fine for most purposes.

Changing the number of iterations and the length of the adaptive period are often desirable. Crucially, the amount of chain thinning should be specified to ensure that users are not saving a large number of MCMC iterations (as this will rapidly fill disk space!). Thinning should be set such that at least 1000 iterations are saved (i.e., `iterations`/`thin` and `thin_hist`). Users are encouraged to pay extra attention to `thin_hist`, which dictates the thinning of the infection history chain, and can generate a very large file if left unchecked.

```
## Distinct filename for each chain
no_chains <- 5
filenames <- paste0(filename, "_",1:no_chains)
chain_path <- sub("par_tab_base.csv","",par_tab_path)
chain_path_real <- paste0(chain_path, "cs1_real/")
chain_path_sim <- paste0(chain_path, "cs1_sim/")

## Create the posterior solving function that will be used in the MCMC framework 
par_tab[par_tab$names == "mu_short","lower_bound"] <- 1
model_func <- create_posterior_func(par_tab=par_tab,
                            titre_dat=titre_dat,
                            strain_isolation_times = strain_isolation_times,
                            version=prior_version) # function in posteriors.R
#> Creating posterior solving function...
#>
```

```
## Generate results in parallel
res <- foreach(x = filenames, .packages = c('serosolver','data.table','plyr')) %dopar% {
  ## Not all random starting conditions return finite likelihood, so for each chain generate random
  ## conditions until we get one with a finite likelihood
  start_prob <- -Inf
  while(!is.finite(start_prob)){
    ## Generating starting antibody kinetics parameters
    start_tab <- generate_start_tab(par_tab)
    
    ## Generate starting infection history
    start_inf <- setup_infection_histories_titre(titre_dat, strain_isolation_times, 
                                                 space=3,titre_cutoff=4)
    start_prob <- sum(model_func(start_tab$values, start_inf)[[1]])
  }
  
  res <- run_MCMC(par_tab = start_tab, 
                  titre_dat = titre_dat,
                  antigenic_map = NULL,
                  strain_isolation_times = strain_isolation_times,
                  start_inf_hist = start_inf, 
                  mcmc_pars = c("iterations"=2000000,"popt"=0.44,"popt_hist"=0.44,
                                "opt_freq"=1000,"thin"=1,"adaptive_period"=500000, 
                                "save_block"=1000, "thin_hist"=100,"hist_sample_prob"=1,
                                "switch_sample"=2, "burnin"=0, "inf_propn"=0.5, 
                                "move_size"=3,"hist_opt"=1,"swap_propn"=0.5,
                                "hist_switch_prob"=0.5,"year_swap_propn"=1),
                  filename = paste0(chain_path_real,x), 
                  CREATE_POSTERIOR_FUNC = create_posterior_func, 
                  version = prior_version)
}
```

# Post-run analyses

Once the MCMC chains are run, `serosolver` provides a number of simple functions to generate standard outputs and MCMC diagnostics. The saved MCMC chains are compatible with the `coda` and `bayesplot` packages, and users are encouraged to use these.

First, read in the MCMC chains. The below function distinguishes between posterior samples for the infection history matrix and for the antibody kinetics parameters. The function searches for all files with the filenames generated by `run_MCMC` in the specified directory, and returns data structures with these concatenated and separated in a list.

```
## Read in the MCMC chains
# Note that `thin` here is in addition to any thinning done during the fitting
# Chain length values in load function need to be consistent with MCMC run
all_chains <- load_mcmc_chains(location=chain_path_real,thin=100,burnin=500000,
                             par_tab=par_tab,unfixed=FALSE,convert_mcmc=TRUE)
#> Chains detected:     5Highest MCMC sample interations: 
#> Chains detected: 
#> X:/Program Files/R/R-3.6.2/library/serosolver/extdata/cs1_real//case_study_1_test_1_infection_histories.csv
#> X:/Program Files/R/R-3.6.2/library/serosolver/extdata/cs1_real//case_study_1_test_2_infection_histories.csv
#> X:/Program Files/R/R-3.6.2/library/serosolver/extdata/cs1_real//case_study_1_test_3_infection_histories.csv
#> X:/Program Files/R/R-3.6.2/library/serosolver/extdata/cs1_real//case_study_1_test_4_infection_histories.csv
#> X:/Program Files/R/R-3.6.2/library/serosolver/extdata/cs1_real//case_study_1_test_5_infection_histories.csv
#> [[1]]
#> [1] 290430
#> 
#> [[2]]
#> [1] 291565
#> 
#> [[3]]
#> [1] 290447
#> 
#> [[4]]
#> [1] 289656
#> 
#> [[5]]
#> [1] 290215
## Alternative, load the included MCMC chains rather than re-running
## data(cs1_chains_real)
## all_chains <- cs1_chains_real

print(summary(all_chains))
#>                   Length Class      Mode   
#> theta_chain       260130 mcmc       numeric
#> inf_chain              5 data.table list   
#> theta_list_chains      5 -none-     list   
#> inf_list_chains        5 -none-     list
```

Chains should then be checked for the usual MCMC diagnostics: \(\hat{R}\) and effective sample size. First, looking at the antibody kinetics parameters:

```
## Get the MCMC chains as a list
list_chains <- all_chains$theta_list_chains
## Look at diagnostics for the free parameters
list_chains1 <- lapply(list_chains, function(x) x[,c("mu","mu_short", "wane",
                                                     "error", "total_infections",
                                                     "lnlike", "prior_prob")])

## Gelman-Rubin diagnostics to assess between-chain convergence for each parameter
print(gelman.diag(as.mcmc.list(list_chains1)))
#> Potential scale reduction factors:
#> 
#>                  Point est. Upper C.I.
#> mu                     1.00       1.01
#> mu_short               1.01       1.04
#> wane                   1.01       1.01
#> error                  1.00       1.00
#> total_infections       1.01       1.02
#> lnlike                 1.01       1.02
#> prior_prob             1.01       1.04
#> 
#> Multivariate psrf
#> 
#> 1.02
gelman.plot(as.mcmc.list(list_chains1))
```

```
## Effective sample size for each parameter
print(effectiveSize(as.mcmc.list(list_chains1)))
#>               mu         mu_short             wane            error 
#>         5050.990         1471.101         1374.883         7055.070 
#> total_infections           lnlike       prior_prob 
#>         1843.337         1262.024         1086.616

## Posterior estimates for each parameter
print(summary(as.mcmc.list(list_chains1)))
#> 
#> Iterations = 1:2001
#> Thinning interval = 1 
#> Number of chains = 5 
#> Sample size per chain = 2001 
#> 
#> 1. Empirical mean and standard deviation for each variable,
#>    plus standard error of the mean:
#> 
#>                        Mean       SD  Naive SE Time-series SE
#> mu                   3.3550  0.06511 0.0006510      0.0017242
#> mu_short             2.5969  0.25167 0.0025161      0.0193919
#> wane                 0.5668  0.06002 0.0006001      0.0018429
#> error                0.7257  0.02537 0.0002536      0.0003147
#> total_infections   145.1587  1.73936 0.0173893      0.0517349
#> lnlike           -1095.8359 12.59805 0.1259490      0.6205704
#> prior_prob        -586.2297 12.20207 0.1219902      0.4048232
#> 
#> 2. Quantiles for each variable:
#> 
#>                        2.5%        25%        50%        75%      97.5%
#> mu                   3.2342     3.3104     3.3525     3.3960     3.4922
#> mu_short             2.0250     2.4507     2.6114     2.7685     3.0359
#> wane                 0.4725     0.5212     0.5595     0.6057     0.6990
#> error                0.6778     0.7082     0.7253     0.7424     0.7784
#> total_infections   142.0000   144.0000   145.0000   146.0000   148.0000
#> lnlike           -1120.8035 -1103.7322 -1096.0770 -1087.7701 -1070.2199
#> prior_prob        -609.1749  -594.4437  -586.5712  -578.4275  -561.2882


## Plot the MCMC trace using the `bayesplot` package
color_scheme_set("viridis")
p_theta_trace <- mcmc_trace(list_chains1)
print(p_theta_trace)
```

and at the infection histories:

```
## Need to adjust x-axis label, as working with quarters not years
x_breaks <- c(strain_isolation_times[seq(1,12,by=2)],8051)
x_labels <- c("Q1-2009","Q3-2009",
              "Q1-2010","Q3-2010",
              "Q1-2011","Q3-2011",
              "Prior")
x_breaks2 <- strain_isolation_times[seq(1,12,by=4)]
x_labels2 <- c("Q1-2009","Q1-2010","Q1-2011")
x_axis <- scale_x_continuous(breaks=x_breaks, labels=x_labels)
x_axis2 <- scale_x_continuous(breaks=x_breaks2, labels=x_labels2)

## Extract infection history chain
inf_chain <- all_chains$inf_chain

## Look at inferred attack rates
## Green shows times that serum samples were taken
p_ar <- plot_attack_rates(inf_chain, titre_dat, strain_isolation_times, pad_chain=TRUE,
                          plot_den = TRUE,prior_pars=list(prior_version=prior_version, 
                                  alpha=par_tab[par_tab$names=="alpha","values"],
                                  beta=par_tab[par_tab$names=="beta","values"])) + x_axis
#> Scale for 'x' is already present. Adding another scale for 'x', which will
#> replace the existing scale.

print(p_ar)
```

```
## Calculate convergence diagnostics and summary statistics on infection histories
## Important to scale all infection estimates by number alive from titre_dat
n_alive <- get_n_alive_group(titre_dat, strain_isolation_times,melt=TRUE)

## This function generates a number of MCMC outputs
ps_infhist <- plot_posteriors_infhist(inf_chain=inf_chain, 
                                      years=strain_isolation_times, 
                                      samples = 100,  
                                      ## Needs to be smaller than length of sampled chain 
                                      n_alive=n_alive)
#> Padding inf chain...
#> Done
#> Calculating by time summaries...
#> Done
#> Calculating by individual summaries...
#> Done


## Posterior mean, median, 95% credible intervals and effective sample size
## on per time attack rates
print(head(ps_infhist[["estimates"]]$by_year))
#>       j group       mean     median lower_quantile upper_quantile
#> 1: 8037     1 0.06270025 0.06188925    0.048859935     0.07491857
#> 2: 8038     1 0.01469070 0.01302932    0.000000000     0.03257329
#> 3: 8039     1 0.01654092 0.01628664    0.009771987     0.02605863
#> 4: 8040     1 0.05514637 0.05537459    0.048859935     0.06188925
#> 5: 8041     1 0.05396715 0.05537459    0.022801303     0.08143322
#> 6: 8042     1 0.03463024 0.03257329    0.000000000     0.09120521
#>    effective_size gelman_point gelman_upper
#> 1:      2704.5912     1.010573     1.027657
#> 2:       680.0930     1.012581     1.033547
#> 3:       482.6676     1.017627     1.043147
#> 4:      4530.5617     1.000146     1.000453
#> 5:      2860.5229     1.003968     1.011721
#> 6:      1596.7841     1.007542     1.021552

## Posterior mean, median, 95% credible intervals and effective sample size
## on per individual total number of infections
print(head(ps_infhist[["estimates"]]$by_indiv))
#>    i     mean median lower_quantile upper_quantile effective_size
#> 1: 1 2.000100      2              2              2      10005.000
#> 2: 2 1.999600      2              2              2      10005.000
#> 3: 3 2.000100      2              2              2      10005.000
#> 4: 4 1.933233      2              1              2       7555.554
#> 5: 6 1.001699      1              1              1      10005.000
#> 6: 7 1.951524      2              1              2       7525.574

## Check for agreement between inferred cumulative infection histories 
## for some individuals
p_indiv_inf_hists <- generate_cumulative_inf_plots(inf_chain, indivs=1:9, pad_chain=FALSE,
                                           nsamp = 100, 
                                           ## Needs to be smaller than length of sampled chain 
                                           strain_isolation_times = strain_isolation_times,
                                           number_col=3)
p1 <- p_indiv_inf_hists[[1]] + x_axis2
## Each subplot shows cumulative number of infections
## over time for an individual. Colours show estimates
## from different MCMC chains.
print(p1)
```

```
## Posterior probability that infections occured at given times per individual
p2 <- p_indiv_inf_hists[[2]] + x_axis2
## Each subplot shows posterior density of infection
## occuring in each quarter for a single individual
print(p2)
```

One of the MCMC chains (chain 2) does not apepar to converge to the same distribution as the others until the second half of MCMC iterations, but the 5 chains do appear to converge to the same posterior distribution and give high effective sample sizes for all parameters. \(\hat{R}\) is also \(<1.1\) for all parameters.

Users may also easily check the inferred antibody landscapes at the time each sample was taken. Black dots show observations, shaded regions and dashed lines show 95% credible intervals on assay measurements (light) and latent true antibody levels (dark) and posterior median.

```
## get_titre_predictions expects only a single MCMC chain, so
## subset for only one chain
chain <- as.data.frame(all_chains$theta_chain)
chain1 <- chain[chain$chain_no == 1,]
inf_chain1 <- inf_chain[inf_chain$chain_no == 1,]
rand_indivs <- c(2,21,36,195)

x_labels <- c("2009-Q1","2009-Q2","2009-Q3","2009-Q4",
             "2010-Q1","2010-Q2","2010-Q3","2010-Q4",
             "2011-Q1","2011-Q2","2011-Q3","2011-Q4")

titre_p <- plot_infection_histories(chain = chain1, 
                                     infection_histories = inf_chain1, 
                                     titre_dat = titre_dat, 
                                     individuals = rand_indivs,
                                     strain_isolation_times = strain_isolation_times,
                                     nsamp = 100, # Needs to be smaller than length of sampled chain 
                                     par_tab = par_tab) +
  scale_x_continuous(expand=c(0,0),labels=x_labels[seq(1,12,by=2)],
                     breaks=strain_isolation_times[seq(1,12,by=2)])
#> Creating model solving function...
#> 
#> Scale for 'x' is already present. Adding another scale for 'x', which will
#> replace the existing scale.

print(titre_p)
```

# Simulation recovery

We finish the vignette by presenting a simulation-recovery experiment to test the ability of the framework to recover known infection histories and antibody kinetics parameters using simulated data that matches the real dataset.

## Extract attack rates from fits

We simulate infection histories and antibody titre data based on the “real” parameters inferred from fitting the model above. First, we extract the maximum posterior probability antibody kinetics parameters and attack rates.

```
## Read in MCMC chains from fitting
all_chains <- load_mcmc_chains(location=chain_path_real,thin=100,burnin=500000,
                               par_tab=par_tab,unfixed=FALSE,convert_mcmc=FALSE)
#> Chains detected:     5Highest MCMC sample interations: 
#> Chains detected: 
#> X:/Program Files/R/R-3.6.2/library/serosolver/extdata/cs1_real//case_study_1_test_1_infection_histories.csv
#> X:/Program Files/R/R-3.6.2/library/serosolver/extdata/cs1_real//case_study_1_test_2_infection_histories.csv
#> X:/Program Files/R/R-3.6.2/library/serosolver/extdata/cs1_real//case_study_1_test_3_infection_histories.csv
#> X:/Program Files/R/R-3.6.2/library/serosolver/extdata/cs1_real//case_study_1_test_4_infection_histories.csv
#> X:/Program Files/R/R-3.6.2/library/serosolver/extdata/cs1_real//case_study_1_test_5_infection_histories.csv
#> [[1]]
#> [1] 290430
#> 
#> [[2]]
#> [1] 291565
#> 
#> [[3]]
#> [1] 290447
#> 
#> [[4]]
#> [1] 289656
#> 
#> [[5]]
#> [1] 290215

## Alternative, load the included MCMC chains rather than re-running
## data(cs1_chains_real_b)
## all_chains <- cs1_chains_real_b

## Find samples that were in both theta and inf hist chains
chain <- all_chains$theta_chain
inf_chain <- all_chains$inf_chain
intersect_samps <- intersect(unique(inf_chain$sampno), unique(chain$sampno))
chain <- chain[chain$sampno %in% intersect_samps,]

## Find the parameter values that gave the highest posterior probability
which_mle <- chain[which.max(chain$lnlike),c("sampno","chain_no")]
mle_theta_pars <- chain[chain$sampno == which_mle$sampno & chain$chain_no == which_mle$chain_no,]

## Store total infections to compare later
mle_total_infs <- mle_theta_pars[,"total_infections"]
mle_theta_pars <- mle_theta_pars[,par_tab$names]
mle_inf_hist <- inf_chain[inf_chain$sampno == which_mle$sampno & 
                            inf_chain$chain_no == which_mle$chain_no,]

## Generate full infection history matrix using provided function
mle_inf_hist <- expand_summary_inf_chain(mle_inf_hist[,c("sampno","j","i","x")])
## Find number of infections per year from this infection history
no_infs <- colSums(mle_inf_hist[,3:ncol(mle_inf_hist)])

## If missing time points in simulated attack rates
if(length(no_infs) < length(strain_isolation_times)){
  diff_lengths <- length(strain_isolation_times) - length(no_infs)
  no_infs <- c(no_infs, rep(0, diff_lengths))
}

## Find attack rate per year
n_alive <- get_n_alive(titre_dat, strain_isolation_times)
attack_rates <- no_infs/n_alive
```

Functions are provided to simulate antibody titre data under a given serosurvey design. The antibody kinetics parameters and attack rates estimated above are used to simulate titres from the model. The `simulate_data` function is well documented, and users should refer to the help file to customise the simulated serosurvey design.

```
set.seed(0)

sim_par_tab <- par_tab
sim_par_tab$values <- as.numeric(mle_theta_pars)
sim_par_tab <- sim_par_tab[sim_par_tab$names != "phi",]
sim_par_tab[sim_par_tab$names %in% c("alpha","beta"),"values"] <- c(1/3,1/3)
sim_par_tab[sim_par_tab$names %in% c("tau","sigma1","sigma2"),"fixed"] <- 1 
sim_par_tab[sim_par_tab$names %in% c("tau","sigma1","sigma2"),"values"] <- 0 
sim_par_tab[sim_par_tab$names == "MAX_TITRE","values"] <- 9 

sampling_times <- seq(2009*resolution + 1, 2012*resolution, by=1)

age_min <- 6*resolution
age_max <- 6*resolution
n_indiv <- length(unique(titre_dat$individual))

dat <- simulate_data(par_tab = sim_par_tab, 
                     n_indiv = n_indiv,
                     buckets = resolution,
                     strain_isolation_times = strain_isolation_times,
                     measured_strains = min(strain_isolation_times),
                     sampling_times = sampling_times, 
                     nsamps = 4, 
                     antigenic_map = NULL, 
                     age_min = age_min,
                     age_max = age_max,
                     attack_rates=attack_rates,
                     repeats = 1)
#> Simulating data


## Inspect simulated antibody titre data and infection histories
sim_titre_dat <- dat[["data"]]
sim_infection_histories <- dat[["infection_histories"]]

## Store total infections to compare later
actual_total_infections <- sum(sim_infection_histories)

## Red lines show times of infection
## Note that x-axis shows quarters (ie. year*4)
plot_data(sim_titre_dat, sim_infection_histories, strain_isolation_times,
          n_indivs = 16,study_design="single_strain")
```

```
sim_ages <- dat[["ages"]]
sim_titre_dat <- merge(sim_titre_dat, sim_ages)
sim_ar <- dat[["attack_rates"]]
```

## Simulation fitting

Once these simulated data have been generated, the work flow becomes exactly the same as with the real data above.

```
filename <- "case_study_1_sim"

## Distinct filename for each chain
no_chains <- 5
filenames <- paste0(filename, "_",1:no_chains)

## Create the posterior solving function that will be used in the MCMC framework 
model_func <- create_posterior_func(par_tab=sim_par_tab,
                                titre_dat=sim_titre_dat,
                                antigenic_map=NULL,
                                strain_isolation_times = strain_isolation_times,
                                version=prior_version) # function in posteriors.R
#> Creating posterior solving function...
#>
```

```
## Generate results in parallel
res <- foreach(x = filenames, .packages = c('serosolver','data.table','plyr')) %dopar% {
  ## Not all random starting conditions return finite likelihood, so for each chain generate random
  ## conditions until we get one with a finite likelihood
  start_prob <- -Inf
  while(!is.finite(start_prob)){
    ## Generate starting values for theta
    start_tab <- generate_start_tab(sim_par_tab)
    ## Generate starting infection history
    start_inf <- setup_infection_histories_titre(sim_titre_dat, strain_isolation_times, 
                                                 space=3,titre_cutoff=4)
    start_prob <- sum(model_func(start_tab$values, start_inf)[[1]])
  }
  
  res <- run_MCMC(par_tab = start_tab, 
                  titre_dat = sim_titre_dat,
                  antigenic_map = NULL,
                  strain_isolation_times = strain_isolation_times,
                  start_inf_hist = start_inf, 
                  mcmc_pars = c("iterations"=1000000,"popt"=0.44,"popt_hist"=0.44,
                                "opt_freq"=1000,"thin"=100,"adaptive_period"=200000, 
                                "save_block"=1000, "thin_hist"=1000,"hist_sample_prob"=0.5,
                                "switch_sample"=2, "burnin"=0, "inf_propn"=1, 
                                "move_size"=3,"hist_opt"=1,"swap_propn"=0.5,
                                "hist_switch_prob"=0.5,"year_swap_propn"=1),
                  filename = paste0(chain_path_sim,x), 
                  CREATE_POSTERIOR_FUNC = create_posterior_func, 
                  version = prior_version)
}
```

## Simulation analysis

MCMC chains should be checked for convergence under the usual diagnostics. We also compare the inferred posterior distributions to the known true parameter values. We see that convergence and between-chain agreement is good and that the model recovers reasonably unbiased estimates for the parameters. Biases in inference may be driven by the contribution of the attack rate prior relative to the contribution of the likelihood (the data). Increasing the number of measured titres (for example, measure titres against 40 viruses rather than 9 or 6 serum samples rather than 4) or using a more informative attack rate prior would help reduce such bias.

```
## Read in the MCMC chains
## Note that `thin` here is in addition to any thinning done during the fitting
sim_all_chains <- load_mcmc_chains(location=chain_path_sim,thin=10,burnin=200000,
                                   par_tab=par_tab,unfixed=FALSE,convert_mcmc=TRUE)
#> Chains detected:     5Highest MCMC sample interations: 
#> Chains detected: 
#> X:/Program Files/R/R-3.6.2/library/serosolver/extdata/cs1_sim//case_study_1_sim_1_infection_histories.csv
#> X:/Program Files/R/R-3.6.2/library/serosolver/extdata/cs1_sim//case_study_1_sim_2_infection_histories.csv
#> X:/Program Files/R/R-3.6.2/library/serosolver/extdata/cs1_sim//case_study_1_sim_3_infection_histories.csv
#> X:/Program Files/R/R-3.6.2/library/serosolver/extdata/cs1_sim//case_study_1_sim_4_infection_histories.csv
#> X:/Program Files/R/R-3.6.2/library/serosolver/extdata/cs1_sim//case_study_1_sim_5_infection_histories.csv
#> [[1]]
#> [1] 13391
#> 
#> [[2]]
#> [1] 13379
#> 
#> [[3]]
#> [1] 13406
#> 
#> [[4]]
#> [1] 13412
#> 
#> [[5]]
#> [1] 13416

## Alternative, load the included MCMC chains rather than re-running
## data(cs1_chains_sim)
## sim_all_chains <- cs1_chains_sim

theta_chain <- sim_all_chains$theta_chain
## Get the MCMC chains as a list
list_chains <- sim_all_chains$theta_list_chains
## Look at diagnostics for the free parameters
list_chains1 <- lapply(list_chains, function(x) x[,c("mu","mu_short","wane",
                                                     "error","total_infections",
                                                     "lnlike","prior_prob")])

## Gelman-Rubin diagnostics and effective sample size
print(gelman.diag(as.mcmc.list(list_chains1)))
#> Potential scale reduction factors:
#> 
#>                  Point est. Upper C.I.
#> mu                     1.01       1.04
#> mu_short               1.00       1.02
#> wane                   1.02       1.07
#> error                  1.00       1.01
#> total_infections       1.01       1.04
#> lnlike                 1.01       1.03
#> prior_prob             1.00       1.01
#> 
#> Multivariate psrf
#> 
#> 1.03
print(effectiveSize(as.mcmc.list(list_chains1)))
#>               mu         mu_short             wane            error 
#>         619.5195         645.0542         457.5725         605.7508 
#> total_infections           lnlike       prior_prob 
#>         562.9334         422.0668         539.8325

melted_theta_chain <- reshape2::melt(as.data.frame(theta_chain), id.vars=c("sampno","chain_no"))
estimated_pars <- c(sim_par_tab[sim_par_tab$fixed == 0,"names"],"total_infections")
melted_theta_chain <- melted_theta_chain[melted_theta_chain$variable %in% estimated_pars,]
colnames(melted_theta_chain)[3] <- "names"

add_row <- data.frame("total_infections",actual_total_infections,0,0.1,0,10000,0,0,1)
colnames(add_row) <- colnames(sim_par_tab)
sim_par_tab1 <- rbind(sim_par_tab, add_row)

ggplot(melted_theta_chain) + 
  geom_density(aes(x=value,fill=as.factor(chain_no)),alpha=0.5) +
  geom_vline(data=sim_par_tab1[sim_par_tab1$fixed == 0,],aes(xintercept=values),linetype="dashed") +
  facet_wrap(~names,scales="free") + 
  theme_classic() +
  theme(legend.position="bottom")
```

Recovery of known attack rates is also reasonably accurate. Again, more titre data or more individuals would improve inferential power if lacking. One particularly reassuring plot is the comparison of known individual cumulative infection histories (the cumulative sum of infections over time for an individual) against the estimated posterior distribution of cumulative infection histories. We see that the 95% credible intervals capture the true cumulative infection histories in almost all cases.

```
## Extract infection history chain
inf_chain <- sim_all_chains$inf_chain

## Look at inferred attack rates
## Green shows times that serum samples were taken
p_ar <- plot_attack_rates(inf_chain, sim_titre_dat, strain_isolation_times, pad_chain=TRUE,
                            plot_den = TRUE,prior_pars=list(prior_version=prior_version, 
                                                          alpha=par_tab[par_tab$names=="alpha","values"],
                                                          beta=par_tab[par_tab$names=="beta","values"]))  +
  geom_point(data=sim_ar,aes(x=year,y=AR),col="purple") + 
  x_axis
#> Scale for 'x' is already present. Adding another scale for 'x', which will
#> replace the existing scale.

print(p_ar)
```

```
## Calculate convergence diagnostics and summary statistics on infection histories
## Important to scale all infection estimates by number alive from titre_dat
sim_n_alive <- get_n_alive_group(sim_titre_dat, strain_isolation_times,melt=TRUE)

## This function generates a number of MCMC outputs
ps_infhist <- plot_posteriors_infhist(inf_chain=inf_chain, 
                                      years=strain_isolation_times, 
                                      n_alive=sim_n_alive,
                                      pad_chain=TRUE)
#> Padding inf chain...
#> Done
#> Calculating by time summaries...
#> Done
#> Calculating by individual summaries...
#> Done

## Check for agreement between inferred cumulative infection histories 
## for some individuals
p_indiv_inf_hists <- generate_cumulative_inf_plots(inf_chain,
                                              indivs=sample(which(rowSums(sim_infection_histories) > 0),9),
                                              pad_chain=TRUE,
                                              real_inf_hist=sim_infection_histories,
                                              strain_isolation_times = strain_isolation_times,
                                              number_col=3)
p1 <- p_indiv_inf_hists[[1]] + x_axis2
## Each subplot shows cumulative number of infections
## over time for an individual. Colours show estimates
## from different MCMC chains.
## Blue lines show true cumulative infection histories
print(p1)
```

```
## Posterior probability that infections occured at given times per individual

## Each subplot shows posterior density of infection
## occuring in each quarter for a single individual
## Vertical red lines show timing of true infections
p2 <- p_indiv_inf_hists[[2]] + x_axis2
print(p2)
```
