# Supplementary material for: An open source tool to infer epidemiological and immunological dynamics from serological data: serosolver
Source: PLoS Comput Biol. 2020 May 4;16(5):e1007840. doi: 10.1371/journal.pcbi.1007840 (PMC7241836; doi:10.1371/journal.pcbi.1007840)
Supplement: S1 Table — (PDF) [file pcbi.1007840.s005.pdf]

Table 1. Summary of infection history priors

Description	Version	Assumption	Summary	Prior on lifetime infections	Prior on augmented attack rates	Use cases
Hyper-prior placed on the probability of infection terms, $\mathbf{\Phi}$	1	Infection status in a given time correlated with infection status of population at that time, but independent of other times.	The prior probability of any individual i becoming infected during time j is $p_{i,j} = \Phi_j$, and all time periods are independent such that each time period j has a unique parameter parameter Φ_j as in Eq 1. These parameters are estimated explicitly and are independent of one another.	binomial	beta-binomial	Where Φ_j is of interest and a distinct user-specified prior is desired for each j, $P(\Phi_j)$. Appropriate when the number of time periods under consideration j is small. Can otherwise lead to poor convergence when j is large. Allows the user full control over the form of $P(\Phi_j)$, which is not possible with other versions.
Beta prior on per-time probability of infection	2	Infection status in a given time correlated with infection status of population at that time, but independent of other times.	Mathematically equivalent to version 1, but $\mathbf{\Phi}$ is integrated out by placing a conjugate beta prior on the probability of infection terms. All infection times j are independent, and users may specify the beta parameters to define the prior distribution.	binomial, $p = \frac{\alpha}{\alpha+\beta}$	beta-binomial with parameters α and β	Unbiased attack rate inference. Reason to assume that individuals are under the same probability of infection process e.g., same location, but require better Markov chain Monte Carlo (MCMC) mixing over inferring all Φ_j independently as in version 1. Appropriate when there are a large number of individuals in the sample, but not necessarily a large amount of antibody data per individual.
Beta prior on per-individual probability of infection	3	Infection status in a given time is independent of infection status of population, but correlated with infection status of that individual at other times.	A beta prior is placed on the probability of a given individual becoming infected in any time period. However, whereas the above priors assumed independence between times but not individuals, this prior assumes independence between individuals but not between times. Infection probabilities are drawn from a single beta distribution.	beta-binomial with parameters α and β	binomial, $p = \frac{\alpha}{\alpha+\beta}$	Unbiased per-individual infection history inference. Reason to assume that individuals are under different infection processes, but share antibody kinetics parameters e.g., different locations or populations. Appropriate with a relatively small number of individuals and large amount of antibody data per individual.
Beta prior on overall probability of infection	4	Infection status correlated to all other infection events i.e., frequent infection in other individuals and in the past suggests more likely to be infected in the future.	A beta prior is placed on the probability of any infection, assuming that infection events are independent both across individuals and time periods.	beta-binomial with parameters α and β	beta-binomial with parameters α and β	Weakly informative priors on both attack rates and lifetime infections are desired, over-dispersed relative to the binomial distribution on all summaries. Appropriate with a small number of individuals and relatively small amount of antibody data per individual, as convergence is slower than under other versions
