# Supplementary material for: An open source tool to infer epidemiological and immunological dynamics from serological data: serosolver
Source: PLoS Comput Biol. 2020 May 4;16(5):e1007840. doi: 10.1371/journal.pcbi.1007840 (PMC7241836; doi:10.1371/journal.pcbi.1007840)
Supplement: S1 Fig — Bars show density histograms of infections from 10,000 simulated infection histories for 100 individuals across 42 infection periods. Red lines show known probability mass function. Plots A, D and G show the prior on the total number of infections per discrete time period j. Plots B, E and H show prior on the total number of lifetime infections per individual. Plots C, F and I show the prior on the cumulative number of infections across 42 time periods for one individual. Black line shows prior median, dark gray region shows 50% credible intervals and light gray region shows 95% credible intervals. Note that priors 1 and 2 are equivalent under these assumptions. (PDF) [file pcbi.1007840.s006.pdf]

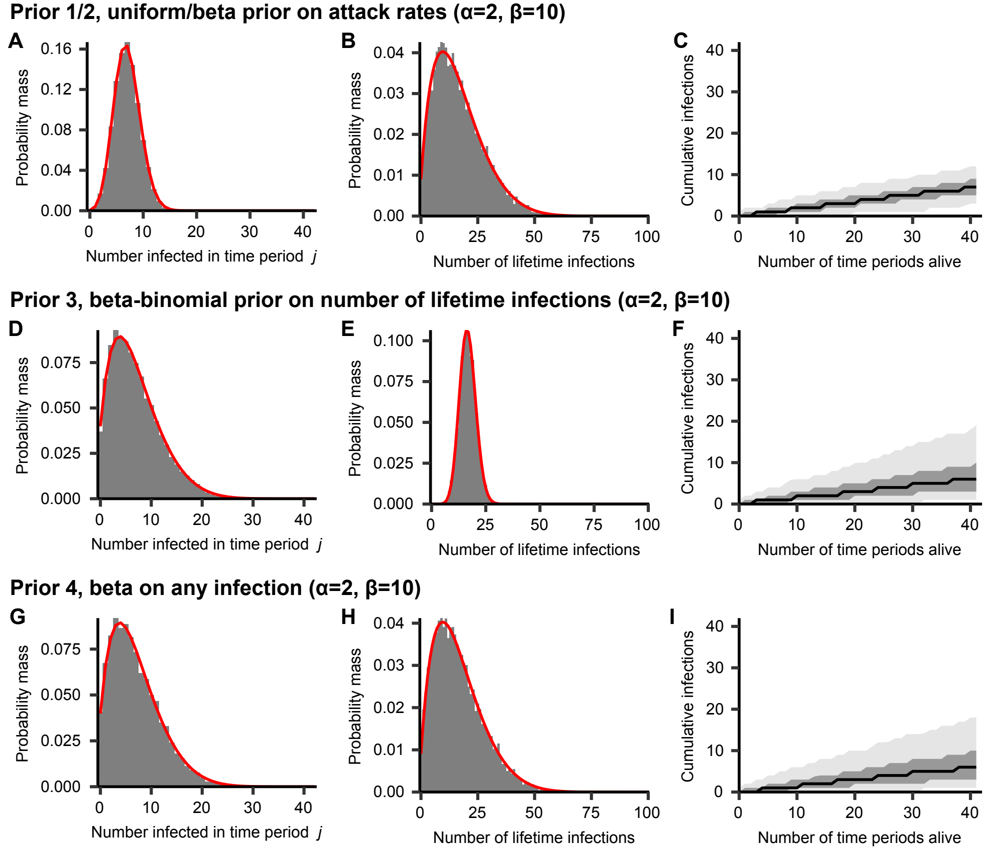
Prior 1/2, uniform/beta prior on attack rates (α=2, β=10)
A
Probability mass
0.16
0.12
0.08
0.04
0.00
0
10
20
30
40
Number infected in time period j
B
Probability mass
0.04
0.03
0.02
0.01
0.00
0
25
50
75
100
Number of lifetime infections
C
Cumulative infections
40
30
20
10
0
0
10
20
30
40
Number of time periods alive
Prior 3, beta-binomial prior on number of lifetime infections (α=2, β=10)
D
Probability mass
0.075
0.050
0.025
0.000
0
10
20
30
40
Number infected in time period j
E
Probability mass
0.100
0.075
0.050
0.025
0.000
0
25
50
75
100
Number of lifetime infections
F
Cumulative infections
40
30
20
10
0
0
10
20
30
40
Number of time periods alive
Prior 4, beta on any infection (α=2, β=10)
G
Probability mass
0.075
0.050
0.025
0.000
0
10
20
30
40
Number infected in time period j
H
Probability mass
0.04
0.03
0.02
0.01
0.00
0
25
50
75
100
Number of lifetime infections
I
Cumulative infections
40
30
20
10
0
0
10
20
30
40
Number of time periods alive
