# Supplementary material for: An open source tool to infer epidemiological and immunological dynamics from serological data: serosolver
Source: PLoS Comput Biol. 2020 May 4;16(5):e1007840. doi: 10.1371/journal.pcbi.1007840 (PMC7241836; doi:10.1371/journal.pcbi.1007840)
Supplement: Attachment — Submitted filename: reviewer_comments_R1.docx [file pcbi.1007840.s007.docx]

We appreciate the thorough and helpful reviews provided by the three reviewers. We believe that addressing their comments and adapting the manuscript for PLOS Computational Biology has strengthened the manuscript immensely. In addition to addressing each specific concern from the reviews, we have made the following improvements to the manuscript:

1. Added a paragraph to the start of the methods and annotated Eq 1 to ease the reader into the full model.
2. Improved the mathematical notation for clarity.
3. Improved Figs 3-5 (now Figs 4-6).
4. Reframed Supporting Information 1 to discuss the general statistical advancements of the method, with the aim of encouraging future adaptation of the framework in addition to the code.

**Reviewer #1**

*1.1 The paper suffers a little from the multiplicity of options in the package, making it hard to keep track of the model structure. For instance, it is a shame that the lengthy and detailed description of the effects of the prior choices on the model inferences are hived off to the SI and could not appear in the main ms. If there is sufficient space (and in an online journal, why would there not be), could some of this useful discussion appear in the main manuscript?*

**[Response 1.1]** We were pleased to see that you appreciated the importance of the infection history priors, as this formed the majority of the theoretical development! Our original aim was to focus the main text on applications and case studies for a more biological audience. However, we agree that these more technical discussion points are suited in PLOS Computational Biology. We have made the following changes to the manuscript:

1. L224 onward: Elaborated on the suitability of each infection history prior for different problems in the methods.
2. L376: Added a results section titled “Infection history priors” comparing the impact of these priors on *a priori* attack rate and total lifetime infection estimates. Also moved a figure from Supporting Information 1 to the main text comparing these priors.

*1.2 Similarly, it was not until my second read of the ms that I understood that time is treated as discrete in the analysis. It may just be that I was inattentive, but I would invite the authors to consider whether the discreteness of time in the model could be made more obvious.*

**[Response 1.2]** We have amended most of the references to time in the introduction and methods sections to “discrete time” to make this clearer.

*1.3 One major omission in my view was the lack of a demonstration of internal validity of their model. Could the authors demonstrate this through for instance plots of individual trajectories with data overlaid, so that the reader may be comforted by the reassurance that the model does adequately characterise the antibody trajectories?*

**[Response 1.3]** Figures 4-6 now all include a panel showing model-predicted latent titres and assay observations against the observed data. In particular, the new subplot in Figure 4 is a key addition. All of the case studies now show the trajectories with data overlaid.

*1.4 L9: There are different initial bases in these two examples: it may be worth commenting upon (10 vs 8).*

**[Response 1.4]** The latter number should have been “1:1280”. However, we have removed this sentence altogether to improve clarity in line with Reviewer #3’s suggestions.

*1.5 L83: Isn’t HK part of China? Might be safer to say “mainland China and HK”.*

**[Response 1.5]** Good point, we have amended as suggested.

*1.6 L128: Zhao et al AJE (10.1093/aje/kwx201) take a superficially similar approach for within season titers, though without many of the important elements of the current paper, such as cross-reactivity. It may be worth mentioning their study somewhere to justify why the current paper is an improvement over existing approaches such as theirs.*

**[Response 1.6]** For PLOS Computational Biology, more discussion of previous methods in this field is needed. We have added the following paragraph to the introduction, including reference to Zhao et al.:

L46: Recently, there have been a number of initiatives to refine the analyses of serological data. Mathematical models that harness the qualitative predictability of the post-exposure antibody response (boosting and subsequent waning of antibody levels) retain much of the information inherent in antibody titres that is otherwise lost using threshold or 4-fold rise metrics, enabling improved inference of unobserved single infections [28]. These methods have been applied in a number of human and wildlife disease systems to understand antibody waning rates and population trends in infection [13, 29-33]. However, dynamical models for antigenically variable pathogen systems with the potential for multiple exposures have been somewhat neglected until recently [27, 34-36]

We have also added the following sentence to introduction paragraph:

L62: Our approach has a number of additions over existing methods designed to support modelling of multi-season, antigenically variable pathogen systems, including the possibility for repeat exposures and cross-reactive antibody responses.

*1.7 L142: Not a new sentence, no capital.*

**[Response 1.7]** Corrected.

*1.8 L169: It would probably be better to give more information on the infection probability parameter priors in the main ms (I realise they are covered in depth in the SI).*

**[Response 1.8]** As discussed above, we have elaborated on these priors in the Methods and Results sections (L224 and L376).

*1.9 L185: Not enough information is given on the format of the data required for the antigenic map. This should probably be elaborated upon.*

**[Response 1.9]** The antigenic map was highlighted by another reviewer, and we have therefore made the following change to the “Data” section to meet both of these comments:

L210: The initial development of *serosolver* focused on influenza A/H3N2, which has circulated in human populations since 1968 and has undergone substantial antigenic evolution over this time [24,39-41]. Fig 1 illustrates how our analytical approach applies to influenza A/H3N2. In *serosolver*, a key but non-essential input to the framework is therefore an antigenic map: a data structure describing the two-dimensional location of viruses in antigenic space that circulated at each time point during the period of interest (specifically, an *x* and *y* coordinate for the strain that circulated in each time period *j*). This map is used to calculate the pairwise antigenic distance between any two viruses (i.e. 𝛿*_m,k_* in the antibody kinetics model, for strains *j* and *k*). Not all disease systems or epidemiological problems require an antigenic map. For example, the map may be considered equivalent to a single point when the pathogen of interest shows no antigenic variability over time. A model with only one circulating antigenic variant may therefore be specified by using a null input for the antigenic map argument.

*1.10 L315: It’s not really ‘essential’ to run multiple chains. It’s often a good idea, yes, but there are many situations where one chain suffices.*

**[Response 1.10]** We have amended with the following text:

L365: Because of the complex nature of these Markov spaces, it is usually important to run multiple chains when using the Metropolis-Hastings MCMC algorithms implemented by *serosolver*. Multiple chains from different starting locations in the space help to detect convergence issues arising, for example, from multi-model posterior distributions.

*1.11 L350: Weren’t the vaccinated individuals vaccinated with the old trivalent vaccine that provided no protection against A-H1N1pdm?*

**[Response 1.11]** Vaccination was a mix of the 2009/10 trivalent seasonal vaccine, 2009 monovalent pandemic vaccine or 2010/11 trivalent seasonal vaccine. The vaccines received before Q4 2009 were indeed the old trivalent seasonal vaccine (there were only 4 doses of monovalent pdm vaccine administered). This point was also raised by Reviewer #3, and we have added the following text:

L456: The pandemic vaccine was available from December 2009, and this increase in inferred boosts from Q1 2010 may therefore capture pandemic vaccine-derived responses. Intuitively, we would expect infection rates to be lower in vaccinated individuals; however, the converse suggests that vaccination caused additional antibody boosting. It is unlikely that these detected boosts represent true infections, as the vaccinated cohort had a larger proportion of older individuals, who likely experienced far lower infection incidence (Fig 4B). Vaccination around the first sampling round was predominantly with the older 2009/10 seasonal vaccination, which was unlikely to elicit a novel antibody response (rather than a memory-derived response) effective against the antigenically dissimilar pandemic virus.

*1.12 L361: The waning is quite different from that observed by Zhao et al (cited above). They found titers waned to baseline sometime between 6 and 12 mo after infection. This seems quite a big deviation, worthy of comment.*

**[Response 1.12]** Based on your comments, we have re-run this case study using the two armed antibody kinetics model (persistent long term boosting and short term boosting that wanes rapidly). The updated waning estimates for the short term responses are now more in line with these previous estimates (only the long term response is left after ~5 months in our results). However, the models are difficult to compare for long term waning, as Zhao et al. model waning of the entire response whereas we have linear waning succeeded by a non-waning long-term response.

Given that these updated results are more in line with these previous estimates, we have not commented on them further. A deeper discussion of the biological implications of these comparative results is beyond the scope of this paper. Instead, we focus on the functionality and flexibility of the package to address such questions.'

*1.13 F3: Would it be possible to enlarge the fonts slightly? It’s quite hard to read.*

**[Response 1.13]** Fonts enlarged to size 8 in line with PLOS figure guidelines.

*1.14 L382: An attack rate of about 100%?? For seasonal influenza? Even a pandemic would not infect that many! The figure clarifies that this occurred several decades before the study. Does it not seem implausible to consider estimating attack rates such a long time ago from HAI titers?*

**[Response 1.14]** We have added a substantial discussion paragraph to address this point. We hope to convey that we acknowledge that this method is still not free from bias in all circumstances, but is a substantial advancement over traditional methods that might be confounded by cross-reactivity and longitudinal antibody dynamics, and one that better reflects appropriate uncertainty in estimate based on the data available

L583: Through using a multi-strain HI assay panel, we estimated historical influenza A/H3N2 attack rates in southern China from 1968 onward. The assumption that the probability of infection was the same for all individuals in the cohort at a given time faithfully captures the expected uncertainty in estimates from times when only few individuals in the cohort were alive. Further data from the Fluscape study with increased strain coverage, number of individuals, and repeated longitudinal serum samples will help improve the precision of these estimates [53]. However, estimates of nearly 100% must be interpreted with caution. In the model, elevated titres to historical strains may only be explained by a fixed amount of homologous and cross-reactive antibody boosting from infection. There are other ways in which relatively high titres might be achieved: different strains may elicit different levels of homologous boosting [53]; systematic bias in the HI assay towards higher titres in particular strains may falsely suggest higher exposure rates [26, 39]; survival of high-immunity individuals may bias the sample; and continual strengthening of titres from asymptomatic exposures should be considered. Alternatively, these estimates may be a true reflection of infection, or at least antibody response, rates that are far higher than previously thought based on traditional methods. Epitope-specific antibody kinetics models combined with state-of-the-art assays may be useful in disentangling the relative contributions of these effects [54, 55].

*1.15 F4: The trajectories don’t look that great, really. Panel C, second from top, right column: there seems to be an undue lack of uncertainty in the mean titer. Is it really epidemiologically plausible that you could be so certain of the true titer at the time of either of the first two observations for this individual?*

**[Response 1.15]** We think that this visually poor fit stems from the distinction between plotting model-predicted latent titres (which are unobserved) and 95% credible intervals on observations. We have updated the trajectories to show both the 95% credible intervals on the latent titres (dark shaded region) and on assay result predictions (light shaded region).

*1.16 F4 cont: Where is the year 2010 on the top panel? I can see a point labelled 2010: is this the mid point of the year? The start of the year? Or something else? Can it be presented less ambiguously? And again if the fonts were a tad larger it would be easier to read this figure.*

**[Response 1.16]** Plot labels were ambiguous, and all plotted values (infection histories and titres) should represent the midpoint of a quarter. The plots and captions have been updated to reflect this.

*1.17 L393: mu -> \mu*

**[Response 1.17]** Corrected

*1.18 F5: panel D: odd choice to use boxplots for a posterior. How to interpret the ‘outliers’ on the boxplot? If the sample had been larger, would they still have appeared?*

**[Response 1.18]** These are boxplots of the posterior medians for each individual. We have clarified this in the figure caption.

*1.19 Supplement 3: Not quite sure why the years are so strange: can elaborate why they are plotted as 8000+?*

**[Response 1.19]** This is an error in labelling from the way the framework converts years to the time resolution of *j* (ie. quarters from the year 2000 are coded as 2000*4=8000+). We have corrected this in the plot labels, and this conversion should be clear from the vignettes.

**Reviewer #2**

*2.1 I doubt anyone without strong quantitative/modeling skills can use this software.*

*Further, the software seems fairly influenza-specific. For instance, the antigenic maps needed as input are as far as I can tell much less commonly used and available for pathogens other than influenza. As such, it seems to me the software is not quite as broadly applicable and usable (yet) as the authors at times seem to suggest.*

**[Response 2.1]** Although some aspects of the software require stronger quantitative skills, we hope that the software will have a good readership at PLOS Computational Biology. This comment was made to a version reviewed at PLOS Biology. We have adapted Supporting Information 1 to describe the model in a general statistical framework, which we hope will encourage adaptation to more disease systems other than influenza.

With regards to the antigenic map: although we developed the package for projects on influenza (and therefore used an antigenic map), the algorithm and model works well for single-strain systems that do not require one, demonstrated by Case Study 1. We have added text to highlight that an antigenic map is not needed, and amended the software to allow users to neglect the antigenic map input (ie. if no antigenic map is specified, the software generates its own null data structure):

L218: **Data**

The *serosolver* package requires a dataset of individual-level log titres. Each individual may have repeat titre measurements from one or more serum sampling times, *t* (i.e. when each serum sample was collected), against viruses that may have circulated at each discrete time period *j* (i.e. when the strain was originally isolated).

The initial development of *serosolver* focused on influenza A/H3N2, which has circulated in human populations since 1968 and has undergone substantial antigenic evolution over this time [24,39-41]. Fig 1 illustrates how our analytical approach applies to influenza A/H3N2. In *serosolver*, a key but non-essential input to the framework is therefore an antigenic map: a data structure describing the two-dimensional location of viruses in antigenic space that circulated at each time point during the period of interest (specifically, an *x* and *y* coordinate for the strain that circulated in each time period *j*). This map is used to calculate the pairwise antigenic distance between any two viruses (i.e. 𝛿*_m,k_* in the antibody kinetics model, for strains *j* and *k*). Not all disease systems or epidemiological problems require an antigenic map. For example, the map may be considered equivalent to a single point when the pathogen of interest shows no antigenic variability over time. A model with only one circulating antigenic variant may therefore be specified by using a null input for the antigenic map argument.

We have also added the additional two sentences to the discussion, second paragraph:

L532: Although our work so far has focused on influenza and therefore uses an antigenic map to specify cross-reactivity between related influenza strains, alternative models to describe cross-reactivity in serological assays could be coded into *serosolver* as part of the antibody kinetics model. For example, independent parameters for the pairwise cross-reactivity of each pathogen in the system could be inferred directly given sufficient.

*2.2 Equation 1 and surrounding text: It is not quite clear to me what the index j (time period) means biologically and how it enters the equation. Can you please explain this some more? (I assume when I see the examples I might understand, but it would be nice to have it explained at first appearance).*

**[Response 2.2]** We have moved this equation and text to the start of the Methods section. We have made the following changes:

1. Annotated Eq 1 for clarity.
2. Added a motivating paragraph to ease the reader into this equation (L79).
3. Added the following paragraph to clarify this particular point:

L106: In each discrete time period, *j*, we assume that there is only one strain that circulates. Reference to *j* therefore refers to both the time period itself and the index of the strain that circulated during that time. Treating time as discrete differs to previous approaches which model infection times as continuous variables [13,35]}. The time resolution of *j* can be set when running *serosolver* depending on the amount of data; using only one possible infection period (*m*=1) is conceptually similar to an analysis of seropositivity, whereas choosing *m* to represent many small intervals of time (e.g. months) becomes conceptually similar to continuous time.

In each discrete time period, *j*, we assume that there is only one strain that circulates. Reference to *j* therefore refers to both the time period itself and the index of the strain that circulated during that time. Treating time as discrete differs to some previous approaches which model infection times as continuous variables [13,35]. The time resolution of *j* can be set when running \code{serosolver} depending on the amount of data; using only one possible infection period (i) is conceptually similar to an analysis of seropositivity, whereas choosing *m* to represent many small intervals of time (e.g. months) becomes conceptually similar to continuous time.

*2.3 Figure 3A: Caption talks about unvaccinated individuals and a black line, figure shows red and blue line and blue belongs to vaccinated. Somehow figure and caption don’t seem to go together?*

**[Response 2.3]** This was an error in the figure caption. The caption has been updated to accurately describe the presented data.

*2.4 Figure 4C talks about ‘latent titres’, I couldn’t find any definition or explanation of that quantity.*

**[Response 2.4]** We have added the following to the “Antibody kinetics model” section at L115:

L115: For a given individual infection history and set of biological parameters, the antibody kinetics model generates a set of expected log titres for that individual against all possible test strains. These antibody titres are observed at only a subset of times for which serum samples are available, and the model-predicted antibody titres across all times are therefore referred to as latent antibody titres.

*2.5 In R, package names are case sensitive. If the package is called serosolver, I suggest the authors stick with this all lower-case spelling and never capitalize it, even at the beginning of a sentence.*

**[Response 2.5]** This is a good justification for a decision that we were torn on. We agree and have changed ‘serosolver’ to be lower case throughout.

**Reviewer #3**

*3.1 First two paragraphs in the Introduction are confusing, and I suggest to revise them for clarity. Reported numbers for antibody titers need the corresponding references. Also, sentences on line 7-9 “Limiting dilutions with higher titres indicate a stronger antibody response…” and on line 22-24 “Because there is a degree of subjectivity in the characterization of a sample being a limiting dilution, a >4-fold difference…” sound confusing. The confusion comes from the use of “limiting dilution” term as there is an assay with this name.*

**[Response 3.1]** We have revised the introduction to improve clarity and flow, particularly for the first two paragraphs. These are shown in the tracked changes version of the revised manuscript. We have added reference to the 2011 WHO “Manual for the laboratory diagnosis and virological surveillance of influenza” in the first paragraph for the numbers and protocols mentioned. We have also removed the term “limiting dilution” throughout.

*3.2 Interestingly, in case Study 1, the model predicted higher pandemic infection rates in vaccinated individuals starting from the fourth quarter of 2009. To explain this result, the authors hypothesized that vaccination boosted the antibody titers that were identified as infection. In Hong Kong, the pandemic H1N1 vaccine became available only in December 2009 [Tarrant et al., Matern Child Health J. 2013 Jan; 17(1): 23–32]. So vaccination probably could not account for the earlier season rise in antibody titers to pandemic strain, as people vaccinated prior to December have been vaccinated with seasonal vaccine which was antigenically different from the pandemic strain. In this case, the predicted higher pandemic infection rates in vaccinated individuals may indicate true pandemic infections. Alternative explanation may be that seasonal vaccine prevented seasonal infections and, thus, prevented corresponding boosting of protective cross-immunity to pandemic strain and*

*increased the chances of being infected.*

**[Response 3.2]** This explanation is possible, but we believe that antibody boosting following vaccination with the pandemic vaccine is more likely. We think that the X-axis alignment of the figure was misleading, and we have updated the figure accordingly. Each axis tick represents the midpoint of that quarter. Therefore, inferred boosting rates are almost identical between vaccinated and unvaccinated individuals up to and including Q4 2009. However, in Q1 2010, where the pandemic vaccine was available, the inferred boosting incidence was far higher in the vaccinated cohort. The vaccinated cohort also had a higher proportion of older individuals, who experienced very low infection incidence in the unvaccinated cohort. It is therefore unlikely that these boosts represent true infections. The updated vaccine administered towards the end of the pandemic may also explain the increased seroresponse rate in vaccinated individuals in Q4 2011. We have added the following text:

L445: We investigated the impact of vaccination status and age on inferred exposure rates, finding differences in exposure rates in vaccinated individuals (n=109) compared to unvaccinated individuals (n=311). Inferred boosting rates were almost identical between vaccinated and unvaccinated individuals up to and including Q4 2009. However, higher overall exposure rates in vaccinated individuals were inferred from Q1 2010 onward (Fig 4A). Additionally, we observed clear differences in age-stratified exposure rates of unvaccinated individuals (Fig 4B), with exposure rates highest among adults (19-64 years old, n=264) and children (<19 years old, n=30), and lowest among the elderly (>64 years old, n=17), confirming previous findings of age-stratified exposure rates during the 2009 pandemic [49]. Some of the inferred infections in Q1 2009 may represent pre-existing baseline titres rather than infections with A/H1N1/pdm09.

L425: The pandemic vaccine was available from December 2009, and this increase in inferred boosts from Q1 2010 may therefore capture pandemic vaccine-derived responses. Intuitively, we would expect infection rates to be lower in vaccinated individuals; however, the converse suggests that vaccination caused additional antibody boosting. It is unlikely that these detected boosts represent true infections, as the vaccinated cohort had a larger proportion of older individuals, who likely experienced far lower infection incidence (Fig 4B). Vaccination around the first sampling round was predominantly with the older 2009/10 seasonal vaccination, which was unlikely to elicit a novel antibody response (rather than a memory-derived response) effective against the antigenically dissimilar pandemic virus.

*3.3 Line 62 extra word “process”*

**[Response 3.3]** Corrected

*3.4 Line 115 “been”->”be”*

**[Response 3.4]** Corrected

*3.5 Line 198 in “must defined” “be” is missing*

**[Response 3.5]** Corrected

*3.6 In Ref [28] pages are missing*

**[Response 3.6]** Reference has been removed with the revised introduction.

*3.7 To refer to their model, authors use “antibody process model” and “antibody kinetics model” through the text. I would suggest to use only one name.*

**[Response 3.7]** Referred to as “antibody kinetics model” throughout.
