# Supplementary material for: An open source tool to infer epidemiological and immunological dynamics from serological data: serosolver
Source: PLoS Comput Biol. 2020 May 4;16(5):e1007840. doi: 10.1371/journal.pcbi.1007840 (PMC7241836; doi:10.1371/journal.pcbi.1007840)
Supplement: Attachment — Submitted filename: reviewer_comments_R2.docx [file pcbi.1007840.s008.docx]

*Author responses shown in blue.*

**Response to Reviewer #1**

Very minor suggestion: I agree with the decision to use lower case consistently with the package name. However you can avoid starting sentences with 'serosolver' by slight rephrasing. For instance:

L58: serosolver takes assay results... -> The serosolver package takes assay results…

Text changed as suggested.

L178: serosolver tracks each individual's infection history as a... -> Each individual's infection history is tracked by serosolver as a…

Changed as suggested.

Might help with the blood pressure of grammar nazi readers.

We have made this change throughout the text as suggested.

L146: Changed to “The *serosolver* model can accommodate antigenically varying strains…”

L167: Changed to “There are additional options in *serosolver* to include strain-specific measurement bias…”

L225: Changed to “We take a Bayesian approach in *serosolver*,”

L240: Changed to “There are four infection history prior options in *serosolver*.”

L305: Changed to “We implemented a custom, adaptive MCMC framework in *serosolver* to…”

L324: Changed to “Functions to test these criteria fall into two broad categories:”

L355: Changed to “Users may create their own likelihood and prior functions on top of those provided by default”

L403: Changed to “The *serosolver* code uses a C++ back-end…”

L489: Changed to “We demonstrate how *serosolver* can be used to reconstruct several features of the epidemiological and immunological dynamics in this cohort.”

L513: Changed to “The *serosolver* package provides a general inference framework to estimate epidemiological and immunological dynamics from serological data.”

L551: Changed to “Prior knowledge on the time of exposure may be incorporated into *serosolver,*”

L562: Changed to “This framework could also be used to inform... “

L607: Changed to “The *serosolver* package provides a generic framework to extract... “

Supplementary Material 1:

Section 4 heading changed to “Priors in serosolver”, and the first sentence has been changed to “There are four options provided in *serosolver* for different infection probability priors.*”*

Supporting Information 2:

Section “Additional boosting assumptions”, first sentence changed to: “There are optional mechanisms included in *serosolver* that account for titre-dependent and strain-dependent boosting.”

**Response to Reviewer #2**

All my concerns from my previous review of the manuscript have been addressed.

It is a well-written, well-organized paper describing nicely documented flexible R package serosolver that will be of interest for those aiming to dissect humoral immune responses to multi-strain pathogens like influenza. I strongly recommend it for publication.

Correction is needed for revised Fig 4B:

Lns 450-453: text refers to the Fig 4B with three age-specific exposure rates, while revised figure shows only two age groups. Corresponding figure legend refers to 3 groups but the green color for age 19-64 on the figure does not match the color indicated in the legend.

The previous Fig 4B incorrectly showed the age-stratified attack rate in the vaccinated population rather than the unvaccinated population (which did not include any individuals >65). We have remade the figure with all 3 age groups included and using the correct (unvaccinated) population. This does not change any of the results described in the main text.

**Response to Reviewer #3**

I previously reviewed this paper for PLoS Biology and suggested that PLoS Comp Bio might be the more suitable journal. The authors addressed all comments I provided on my previous review, I have no further ones.

One general comment, mainly meant for the editor: Instead of this being labeled a 'research article', it might be better to classify it as a 'software article' (or whatever terminology the journal uses).

We are open to the editor’s suggestions on this comment. We feel that either option is appropriate, as our paper is intended to describe the underlying methods in detail as well as the package itself.
